# Supplementary material for: 3D reconstruction of skin and spatial mapping of immune cell density, vascular distance and effects of sun exposure and aging
Source: Commun Biol. 2023 Jul 19;6:718. doi: 10.1038/s42003-023-04991-z (PMC10356782; doi:10.1038/s42003-023-04991-z)
Supplement: Supplementary file 2 — Supplementary Information [file 42003_2023_4991_MOESM2_ESM.pdf]

Supplementary Figures and Tables

Ghose, Ju *et al.* 3D reconstruction of skin and spatial mapping of immune cell density, vascular distance and effects of sun exposure and aging

Supplementary Figure 1 - Layout of samples in each tissue section on a representation of a glass slide (shown as virtual H&E images). Samples ranged in size between 2 mm – 7 mm (region 8 highlighted for reference)

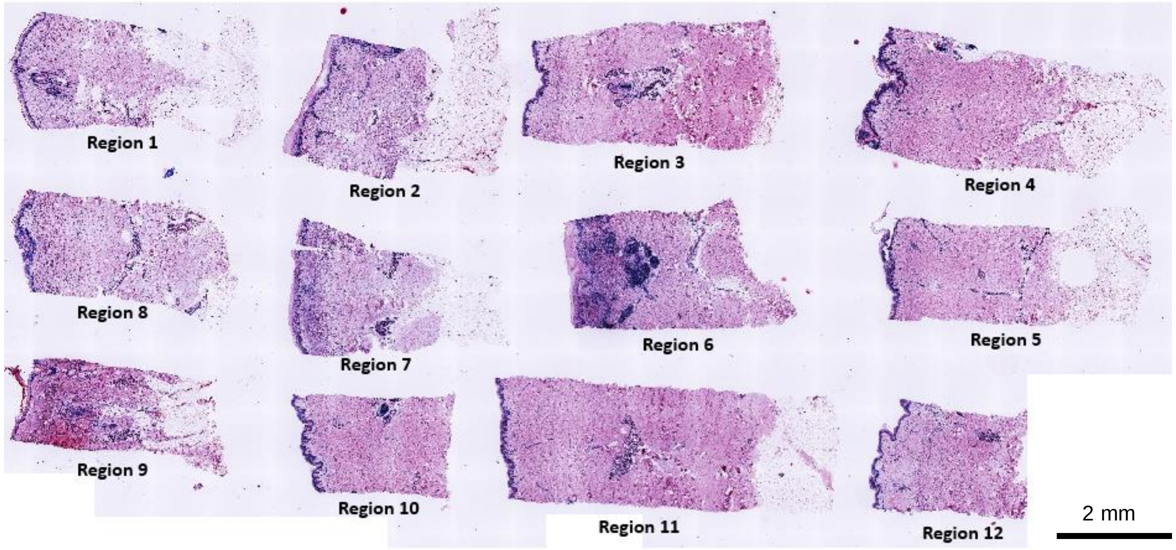

Supplementary Figure 2 - Anatomical locations for the skin biopsy for females and males on the HuBMAP portal Exploration User Interface - [HuBMAP CCF Exploration User Interface (CCF-EUI) (hubmapconsortium.org)]

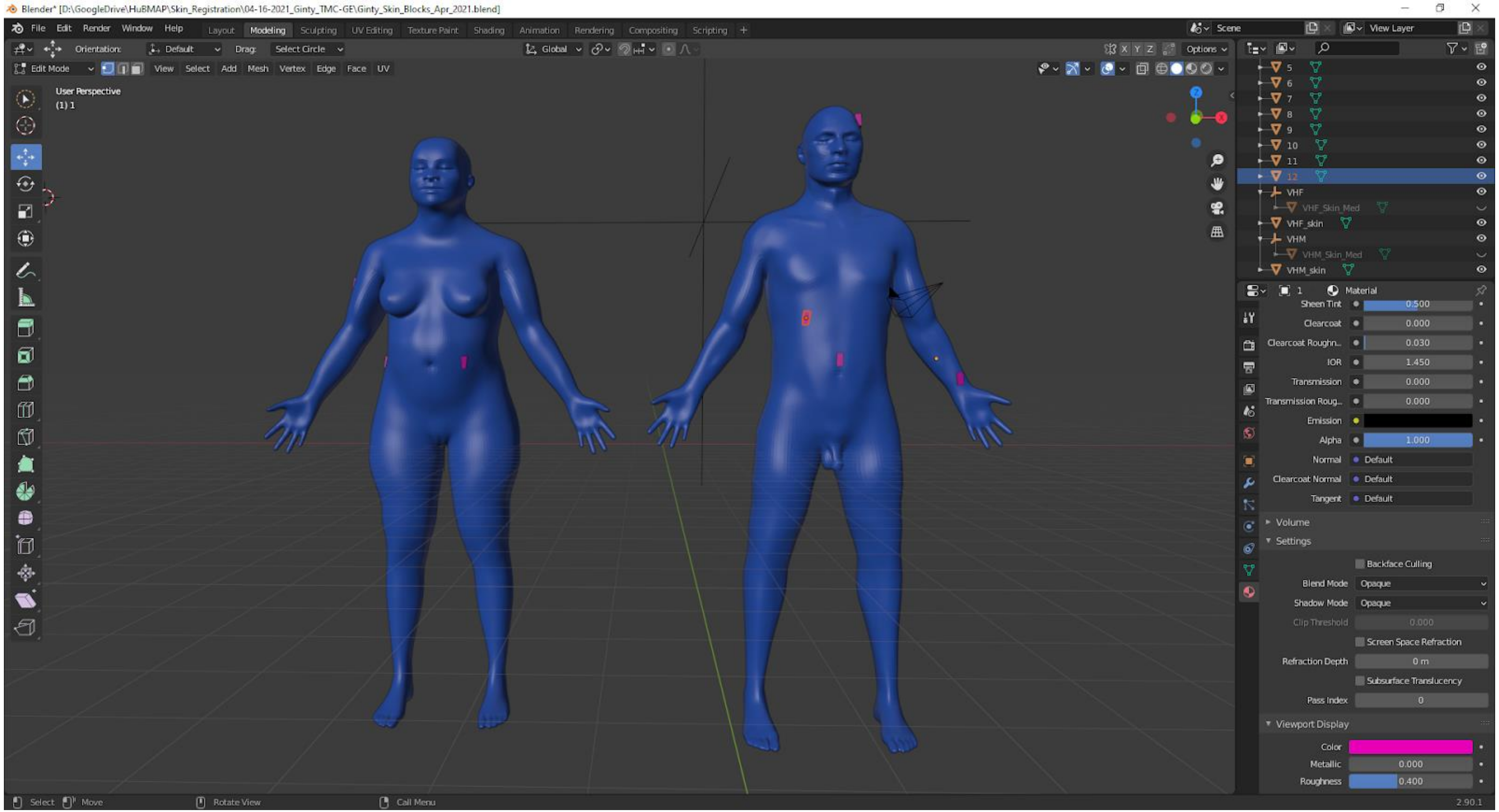

Supplementary Figure 3 - A subset of the skin anatomical structures, cell types and biomarkers (ASCT+B) captured from the reporting tool. Anatomical structures are shown as red circles, unique cell types in blue, and protein biomarkers in green. Interactive visualization of all cell types and markers for all organs is available at [ASCT+B Reporter (hubmapconsortium.github.io)]() and specifically for skin at [ASCT+B Skin Report]().

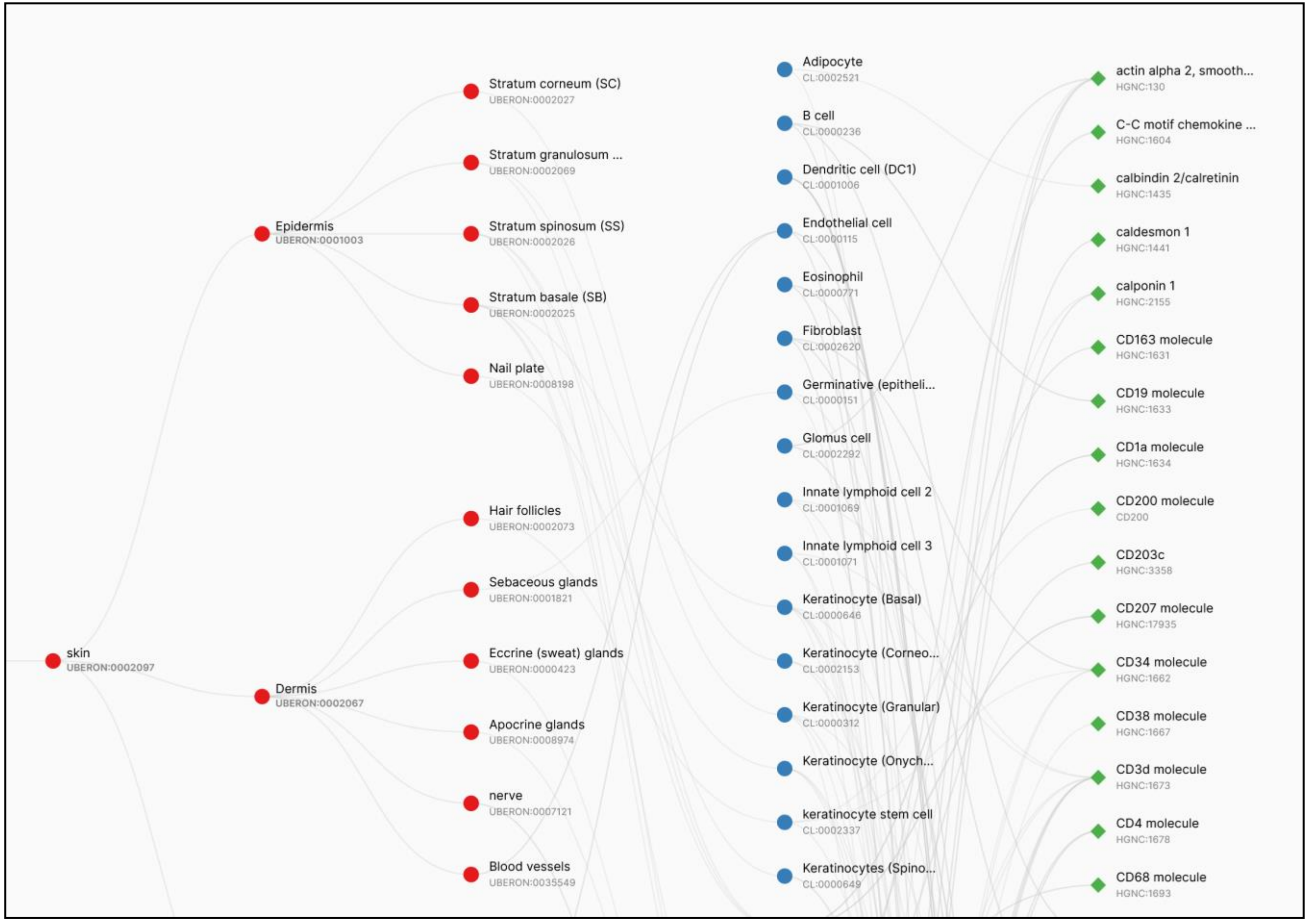

Supplementary Figure 4 – Micro CT imaging workflow. a) Samples were embedded in a single paraffin block, which then underwent micro CT imaging prior to block sectioning; b) shows an example section with AF images; c) the same section but shown as a virtual H&E and each sample is annotated as region 1, 2, etc.; d-e) zoomed in example of the Micro CT slice for region 6; f) Corresponding virtual H&E section for region 6.

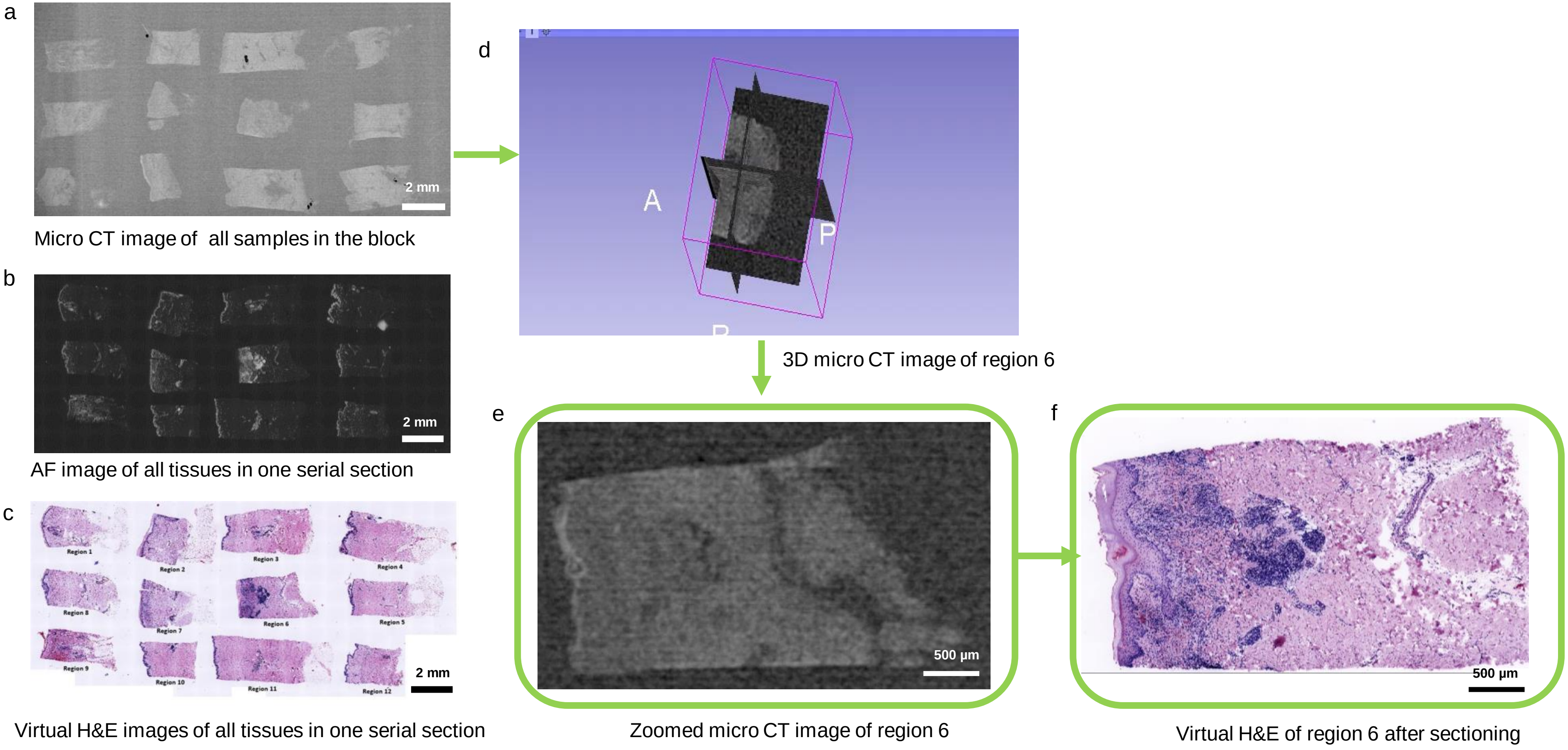

Supplementary Figure 5 - Cell segmentation using an encoder-decoder based deep learning (DL) model. A hybrid supervised and unsupervised model where a supervised DL model was used for DAPI/nuclei segmentation. First, an encoder-decoder based deep learning (DL) model was trained on a small sample (194 DAPI image patches selected from 30 images from 10 patients) of manually annotated nuclei created using the annotation function in QuPATH. Multiscale Laplacian of Gaussian (LOG) was introduced along with the DAPI images as separate channels to our encoder-decoder based DL model. The LOG feature detects blob like structures (which correspond to nuclei shape and boundary) in the DAPI images, and thereby provides contextual information to the DL model. Use of multiple channels allowed us to train an accurate DL model from a small sample of the manually annotated DAPI images. The depth of the encoder-decoder DL model was set to 4 and binary cross entropy was used as a loss function.

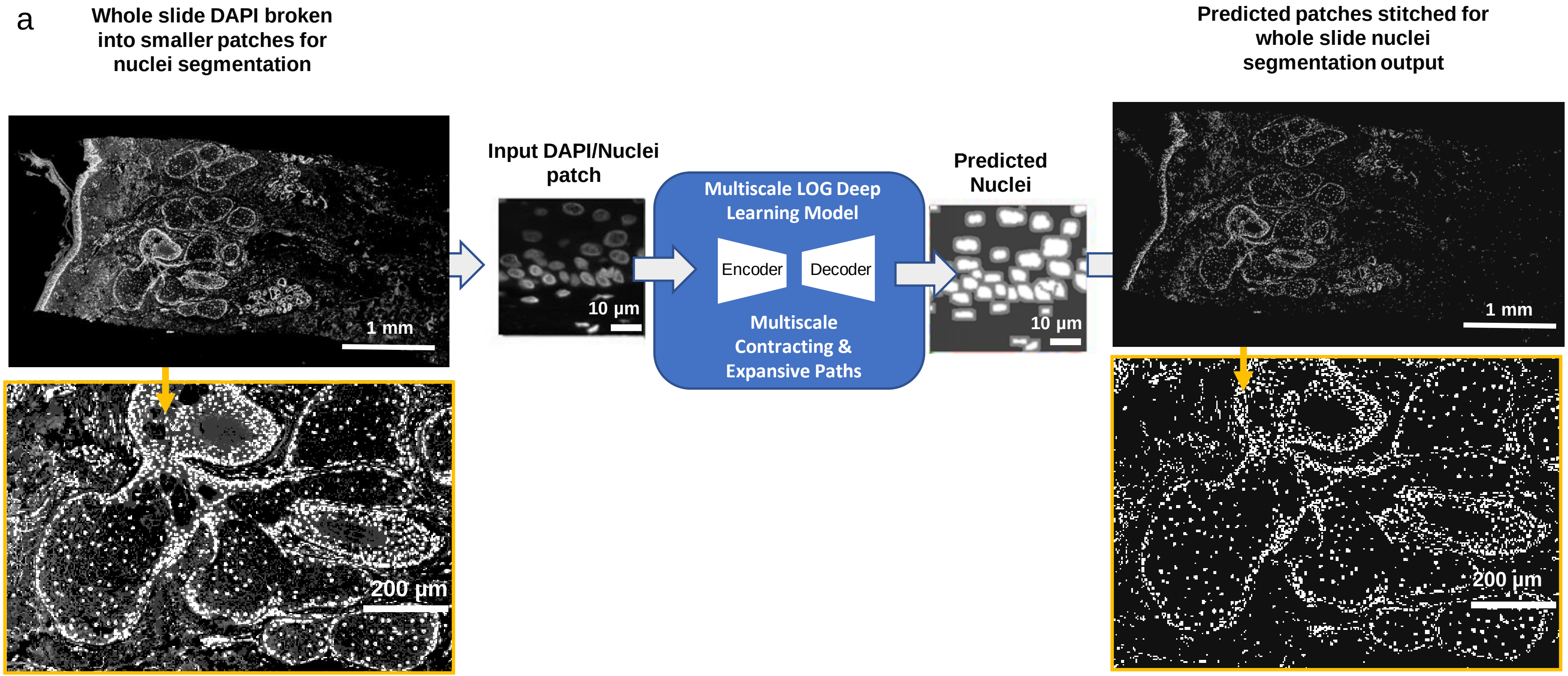

Supplementary Figure 5 - Supervised Cell Segmentation and Classification Model. An unsupervised GMM was used for automatic probabilistic segmentation of immune cell types: T killer (CD8), T reg (FOXP3), T helper (CD4), macrophages (CD68), as well as endothelial cells (CD31), markers of proliferation (Ki67), DNA damage (p53), and DNA repair (DDB2). GMM with two clusters was used to obtain a probabilistic segmentation of the cell-type and functional markers and background. Pixels with high signal for cell type and functional markers were automatically assigned high probability values in one cluster (positive class) and pixels with low signal intensity were assigned high probability values in the second cluster (background – negative class). Union of probabilities obtained from the positive class of GMM model and nuclei segmentation (from the DL model) were then fused. Probability values were used to automatically scale (between 0-1) and quantify positive cells (i.e., for each marker of interest) and determine the percentage overlap between the markers and the segmented nuclei. Low percentage overlap was further used to remove imaging artifacts, debris, and cells with low/background marker intensity.

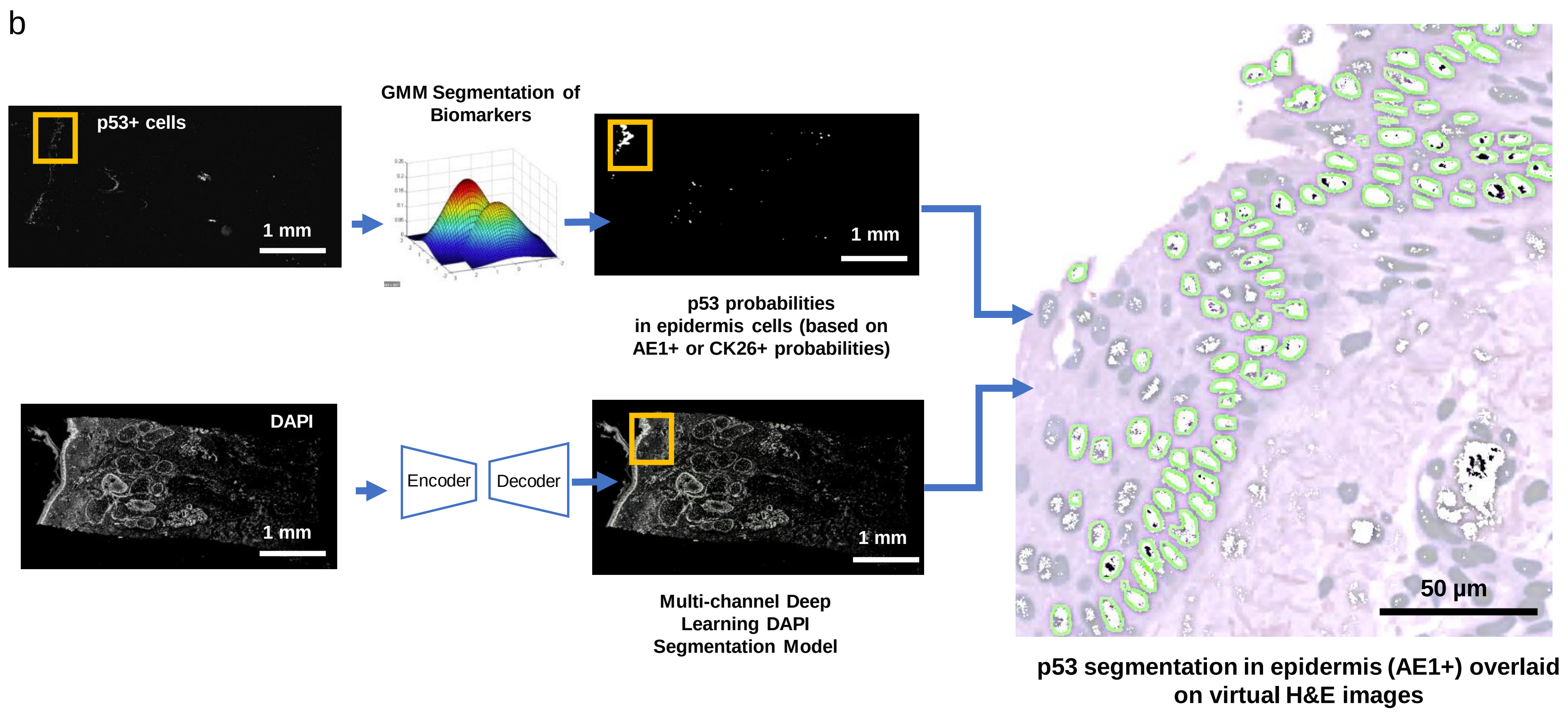

Supplementary Figure 6 - Normalized 3D immune cell counts analyzed by age and sun exposure in the entire sample (dermis and epidermis) (a-g) and correlations (h); There was a non-significant trend for a positive relationship between T Helper/T Killer cell counts and age (p=0.01, adj. p=0.07) (see panel h).

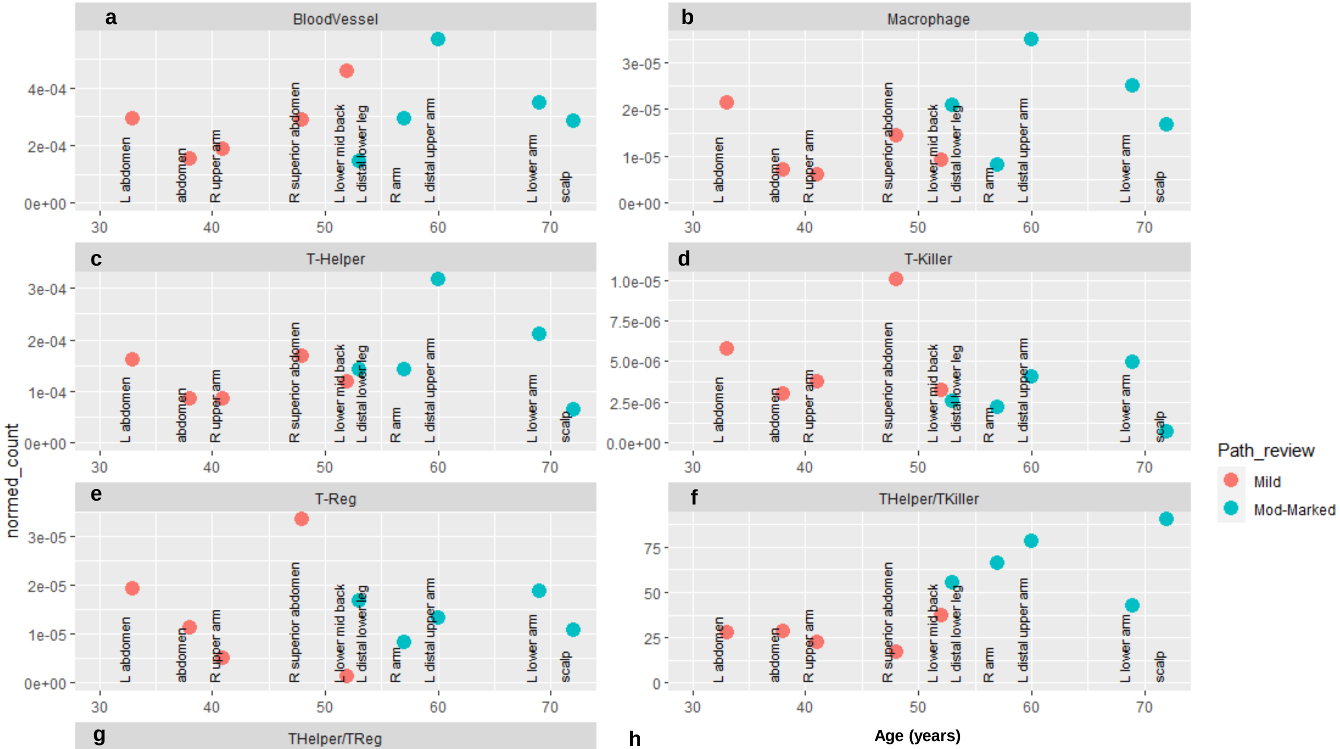

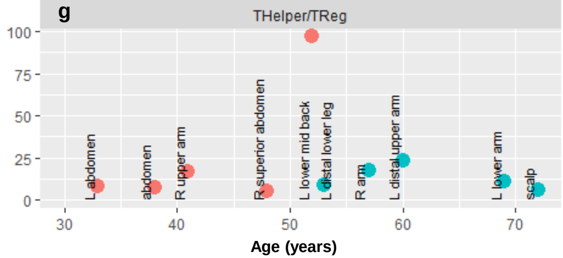

h

	Spearman correlation with age (Test statistics, p-value, BH adj p-value)	Wilcoxon test with UV exposure Test statistics (p-value, BH adj p-value)
**Blood Vessel**	0.35 (S=108, p=0.33, adj.p=0.58)	W=108 (p=0.69, adj.p=0.8)
**Macrophage**	0.44 (S=92, p=0.2, adj.p=0.58)	W=92 (p=0.15, adj.p=0.51)
**T Helper**	0.14 (S=142, p=0.71, adj.p=0.76)	W=142 (p=0.55, adj.p=0.8)
**T Killer**	-0.39 (S=230, p=0.26, adj.p=0.58)	W=230 (p=0.22, adj.p=0.51)
**T Reg**	-0.12 (S=184, p=0.76, adj.p=0.76)	W=184 (p=1, adj.p=1)
**T Helper/T Killer**	0.82 (S=30, p=0.01, adj.p=0.07)	W=30 (p=0.01, adj.p=0.07)
**T Helper/T Reg**	0.18 (S=136, p=0.63, adj.p=0.76)	W=136 (p=0.69, adj.p=0.8)

Supplementary Figure 7 - Immune cell counts within 100 µm of endothelial cells and correlations with age/skin exposure to UV (a-d) and correlations (e); (iii) There was a trend for an inverse relationship between T Killer cell counts within 100 µm of endothelial cells and age (adj.p=0.08) (see panel e)

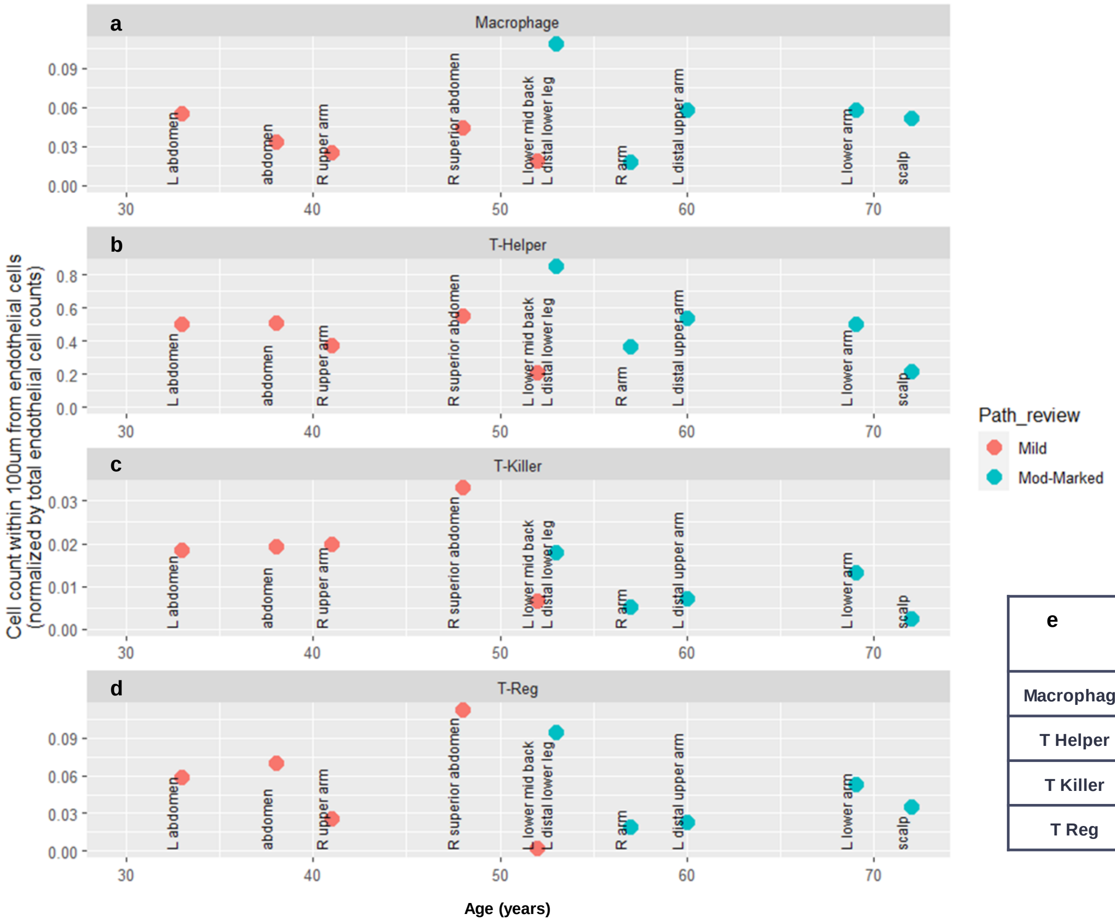

e	Spearman correlation with age (Test statistics, p-value, BH adj p-value)	Wilcoxon test with UV exposure Test statistics (p-value, BH adj p-value)
**Macrophage**	0.27 (S=120, p=0.45, adj.p=0.6)	W=120 (p=0.22, adj.p=0.44)
**T Helper**	-0.14 (S=188, p=0.71, adj.p=0.71)	W=188 (p=0.84, adj.p=0.84)
**T Killer**	-0.73 (S=286, p=0.02, adj.p=0.08)	W=286 (p=0.06, adj.p=0.22)
**T Reg**	-0.32 (S=218, p=0.37, adj.p=0.6)	W=218 (p=0.69, adj.p=0.84)

Supplementary Figure 8 - p53 and Ki67 positive cells deep in the epidermis are associated with hair follicle regions: Region 1 (scalp, marked sun exposure, 72 years) shows a higher distribution of p53 and Ki67 positive cells associated with hair follicle cells and up to 1600 µm deep with a reference virtual H&E section from the same sample with a hair follicle highlighted. For interactive visualization go to: https://hubmapconsortium.github.io/vccf-visualization-release/html/epidermis_entire/epidermis_region_1.html

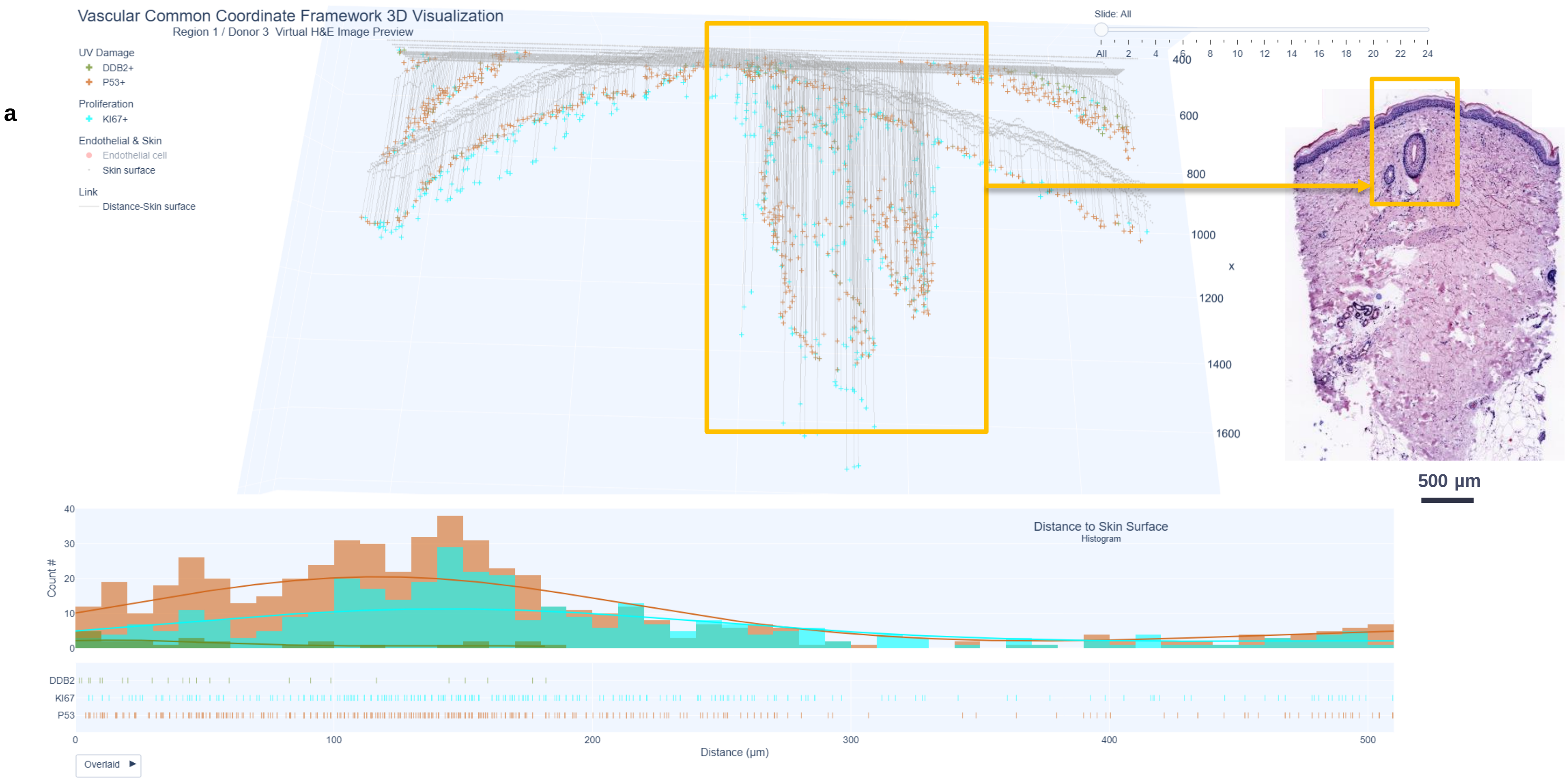

Supplementary Figure 8 – Region 2 (lower mid back, mild sun exposure, 52 years) shows a higher distribution of p53 and Ki67 positive cells associated with hair follicle cells and up to 1600 µm deep and a reference virtual H&E section from the same sample with a hair follicle along the edge is highlighted. For interactive visualization go to: https://hubmapconsortium.github.io/vccf-visualization-release/html/epidermis_entire/epidermis_region_2.html

b

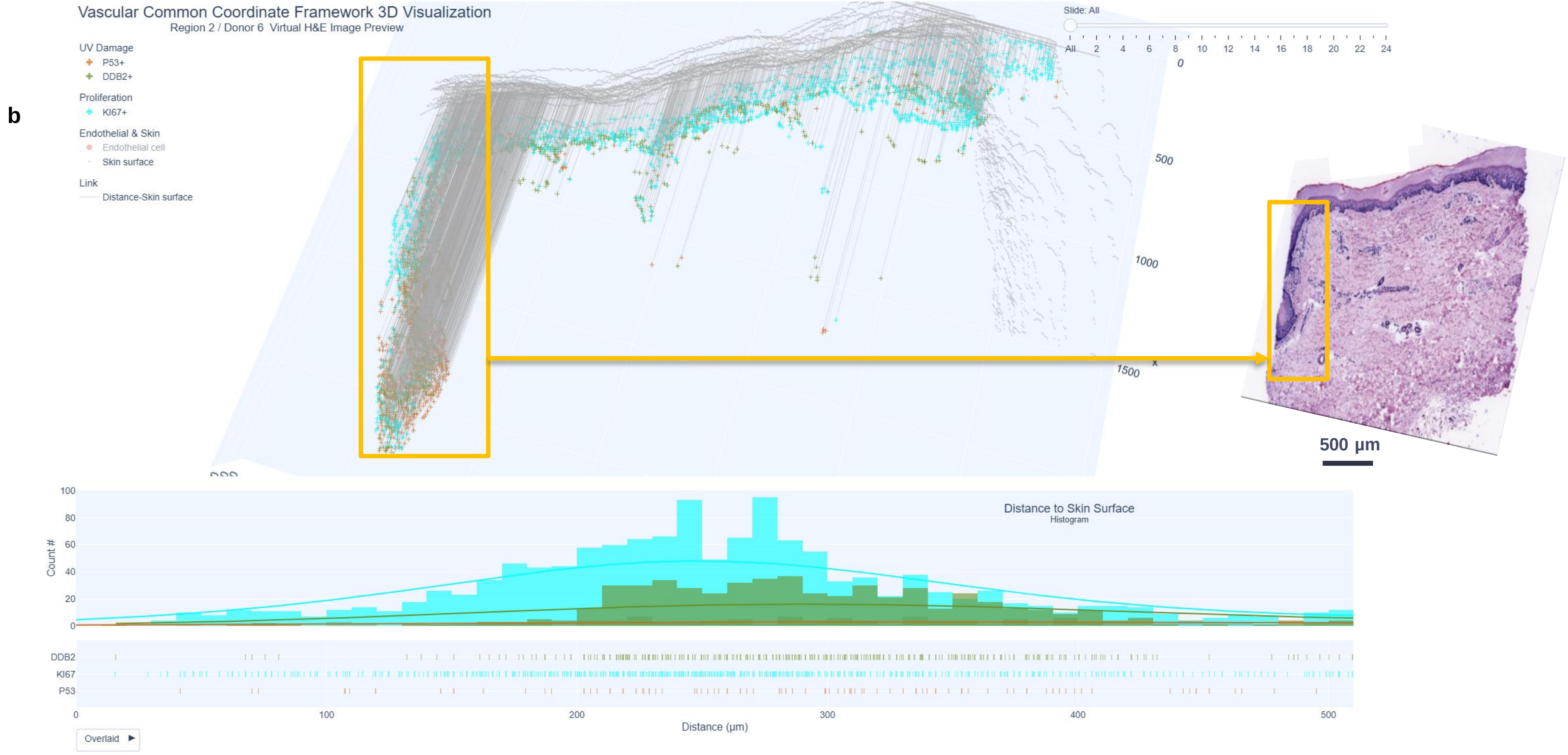

Supplementary Figure 8 -. Region 9 (upper arm, marked sun exposure, 60 years) shows a higher distribution of p53 and Ki67 positive cells associated with hair follicle cells and up to 1600 µm deep with a reference virtual H&E section from the same sample and highlighted hair follicle. For interactive visualization go to: https://hubmapconsortium.github.io/vccf-visualization-release/html/epidermis_entire/epidermis_region_9.html

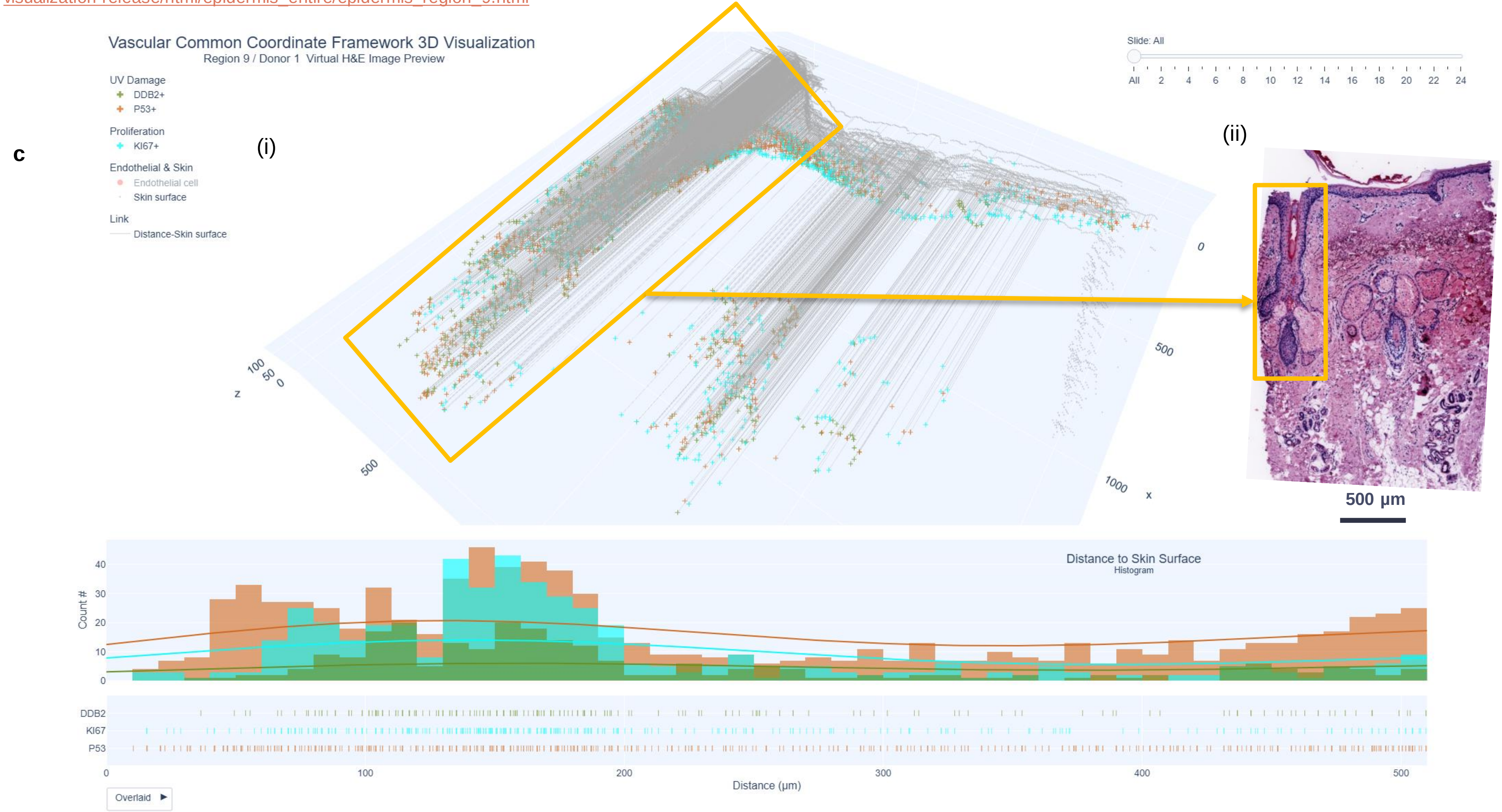

Supplementary Figure 9 - Normalized epidermis (AE1/stratum basale layer) cell counts for DNA damage (DDB2 and p53) and proliferation (Ki67) (a-d); (e) correlations with age and skin exposure to UV. There was a significant inverse correlation between DDB2 positive cells and age (adj. p=0.05) (see panel e).

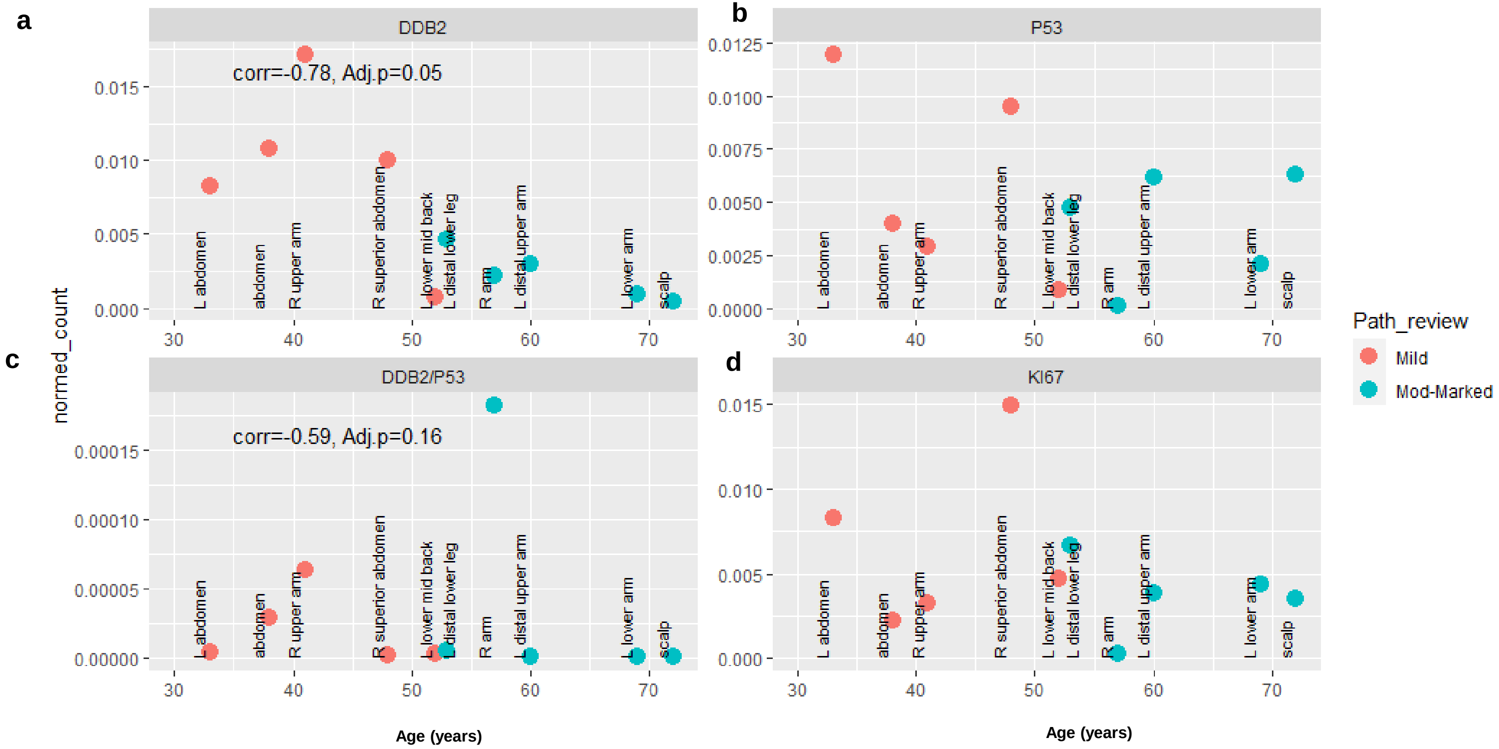

e

	Spearman correlation with age (Test statistics, p-value, BH adj p-value)	Wilcoxon test with UV exposure Test statistics (p-value, BH adj p-value)
**DDB2**	-0.78 (S=294, p=0.01, adj.p=0.05)*	W=294 (p=0.1, adj.p=0.38)
**P53**	-0.22 (S=202, p=0.54, adj.p=0.58)	W=202 (p=0.69, adj.p=0.69)
**DDB2/P53**	-0.59 (S=262, p=0.08, adj.p=0.16)	W=262 (p=0.42, adj.p=0.69)
**KI67**	-0.2 (S=198, p=0.58, adj.p=0.58)	W=198 (p=0.55, adj.p=0.69)

Supplementary Figure 9 - Normalized epidermis (CK26/entire epidermis) cell counts for DNA damage (DDB2 and p53) and proliferation (Ki67) (f-i); (J) correlations with age and skin exposure to UV. There was a significant inverse correlation between DDB2 positive cells and age (adj. p=0.04) (see panel j).

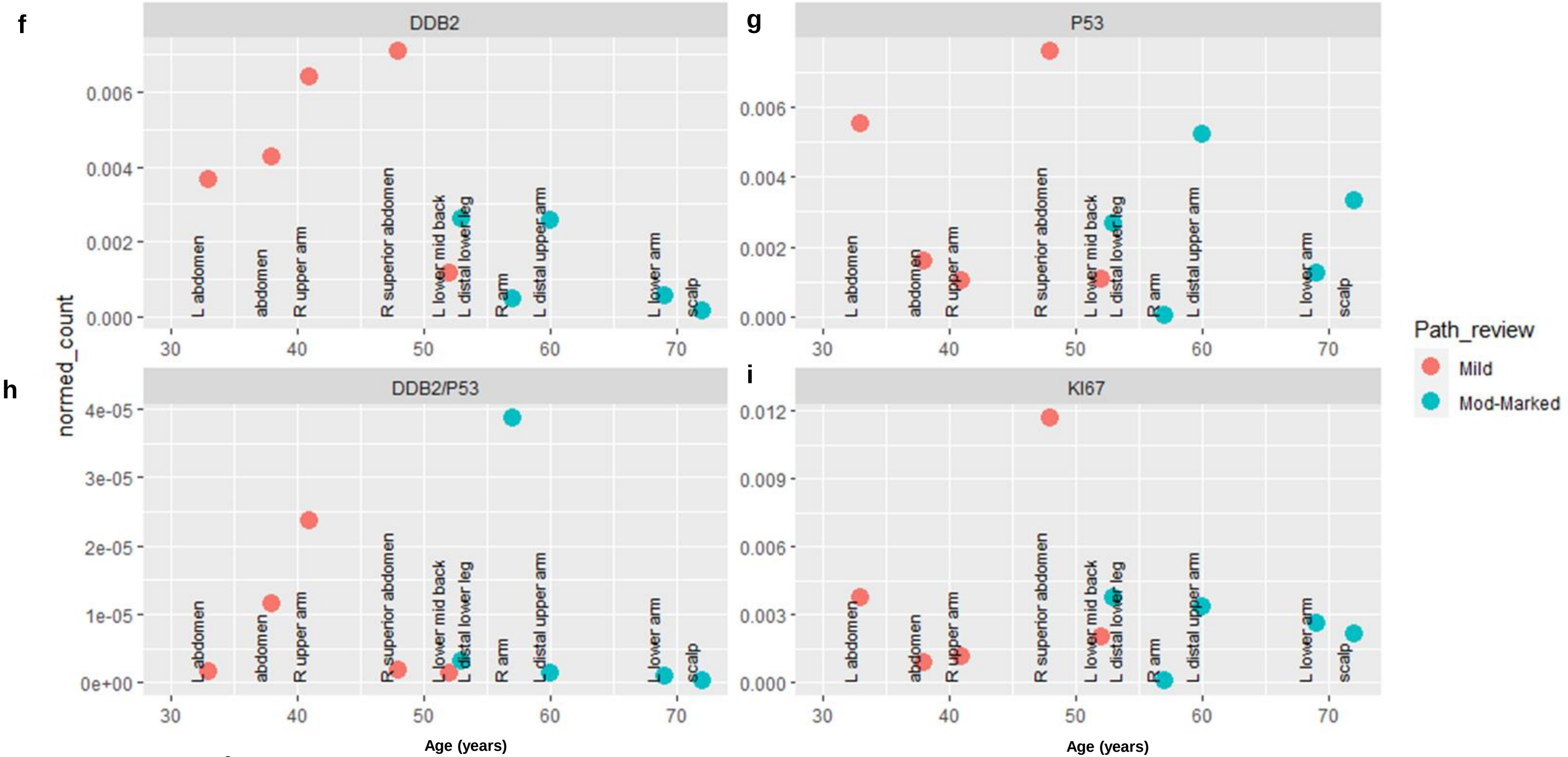

j

	Spearman correlation with age (Test statistics, p-value, BH adj p-value)	Wilcoxon test with UV exposure Test statistics (p-value, BH adj p-value)
**DDB2**	-0.79 (S=296, p=0.01, adj.p=0.04)*	W=296 (p=0.03, adj.p=0.13)
**P53**	-0.12 (S=184, p=0.76, adj.p=0.86)	W=184 (p=0.84, adj.p=1)
**DDB2/P53**	-0.53 (S=252, p=0.12, adj.p=0.25)	W=252 (p=0.55, adj.p=1)
**KI67**	-0.07 (S=176, p=0.86, adj.p=0.86)	W=176 (p=1, adj.p=1)

Supplementary Figure 10 – a Virtual H&E of region 11 from 41-year-old donor (upper arm) with mild sun exposure; **b** Region 7 from 69-year-old donor (lower arm) with marked sun exposure based on pathologist review and standardized assessment criteria.

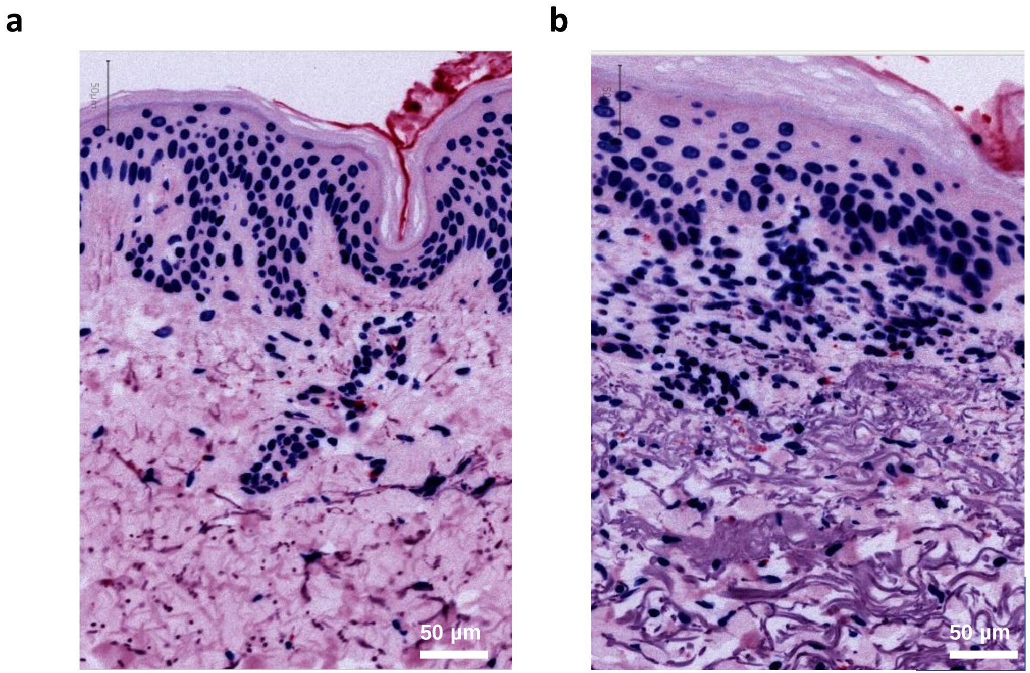

Supplementary Figure 11 - Example images for pan cytokeratin PCK26 in earlier validation studies and in the multiplexed skin study. As described in methods, a multi-step process was utilized and is available on protocols.io Cell DIVE™ Platform | Antibody Characterization for Multiplexing (protocols.io). A multi-organ slide (Pantomics MTU391) containing tumor and corresponding uninvolved tissues as controls is used for screening antibodies and testing dye conjugates and concentrations. Example images for PCK26 are shown from prostate and skin.

a

Pan Cytokeratin - PCK26 - KRT 1, 5, 6A, 6B, 6C, 8 (Sigma C5992).

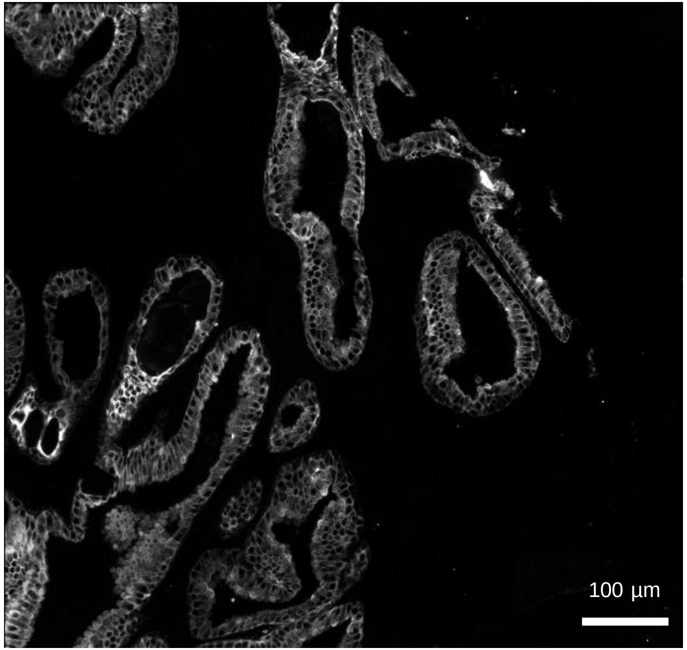

Normal prostate 3.3 D/P Cy3 at 2.5 µg/mL

Region of interest with Pan CK26 staining in multiplexed skin sample

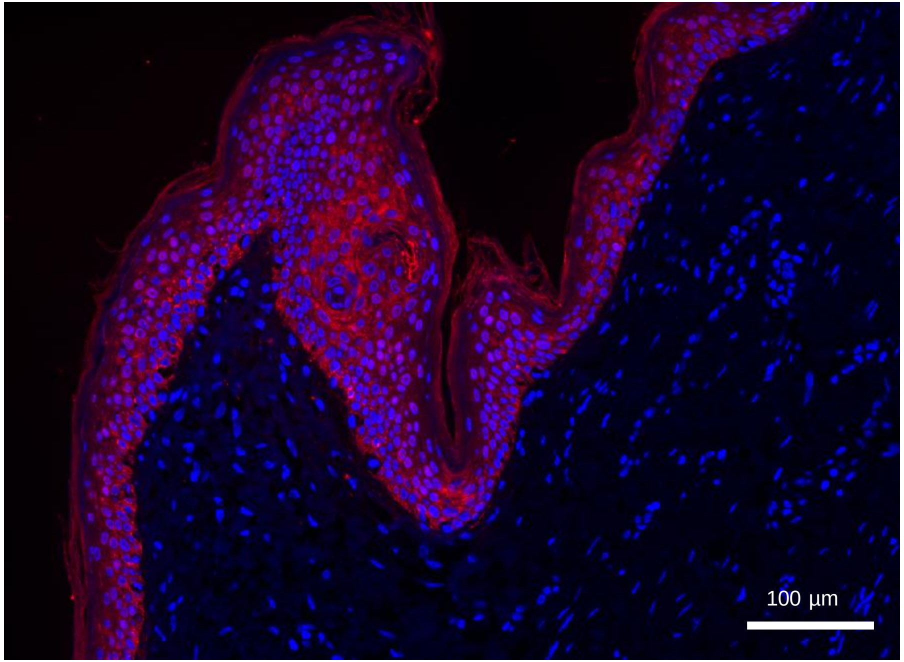

DAPI - Blue, PCK26 - Red

Supplementary Figure 11 - Example images for pan cytokeratin AE1 in earlier validation studies and in the multiplexed skin study. As described in methods, a multi-step process was utilized and is available on protocols.io [Cell DIVE™ Platform | Antibody Characterization for Multiplexing (protocols.io)]. A multi-organ slide (Pantomics MTU391) containing tumor and corresponding uninvolved tissues as controls is used for screening antibodies and testing dye conjugates and concentrations. Example images for AE1 are from normal skin.

b

Pan Cytokeratin AE1 - KRT10, 14, 15, 16, 19 (Thermo Fisher 14-9001)

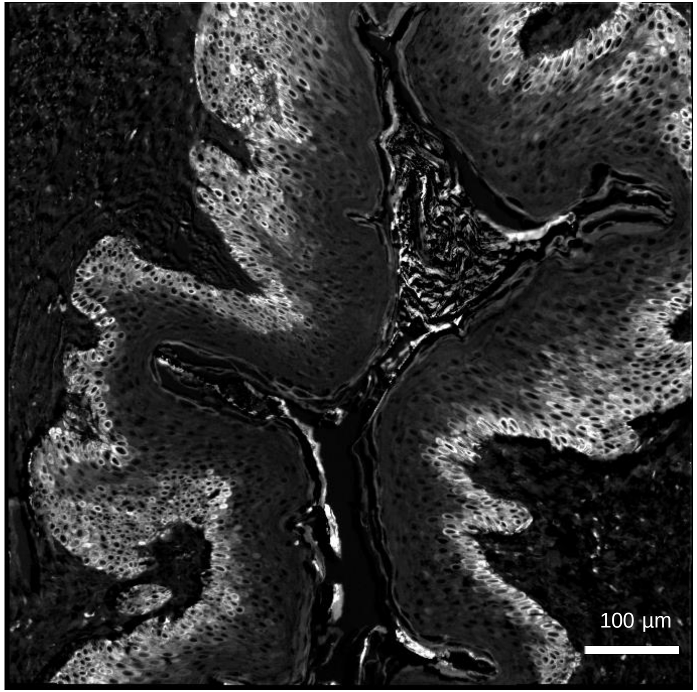

Normal skin 2.7 D/P Cy3 at 5 µg/mL

Region of interest with AE1 staining in multiplexed skin sample

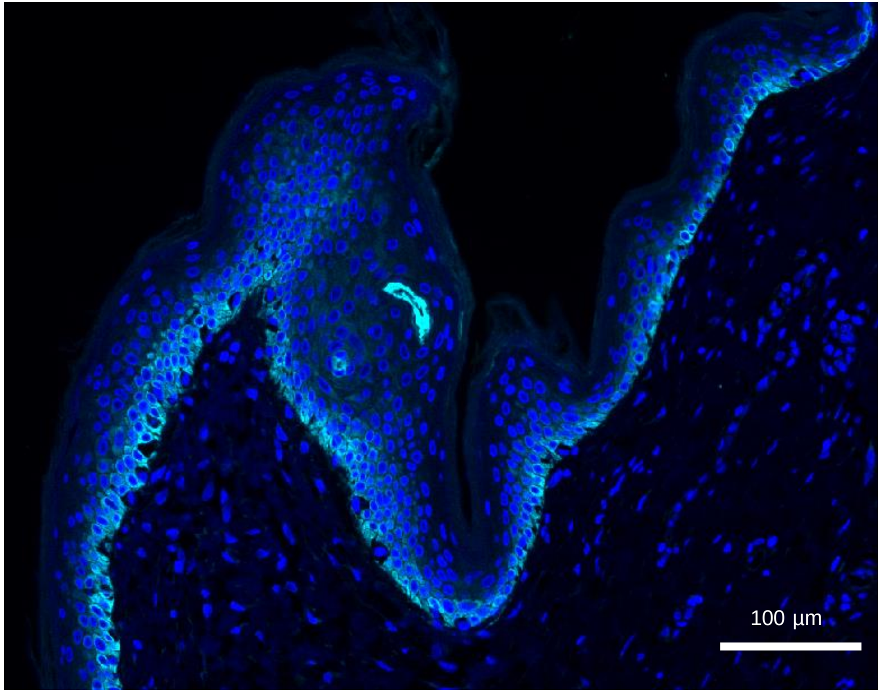

DAPI – Blue, AE1 - Cyan

Supplementary Figure 11b (continued) – Side by side comparison of Pan CK26 (red), AE1 (cyan) and both images combined as an overlay. CK26 stained the entire epidermis region and AE1 was more localized to the lower region of the epidermis (stratum basale).

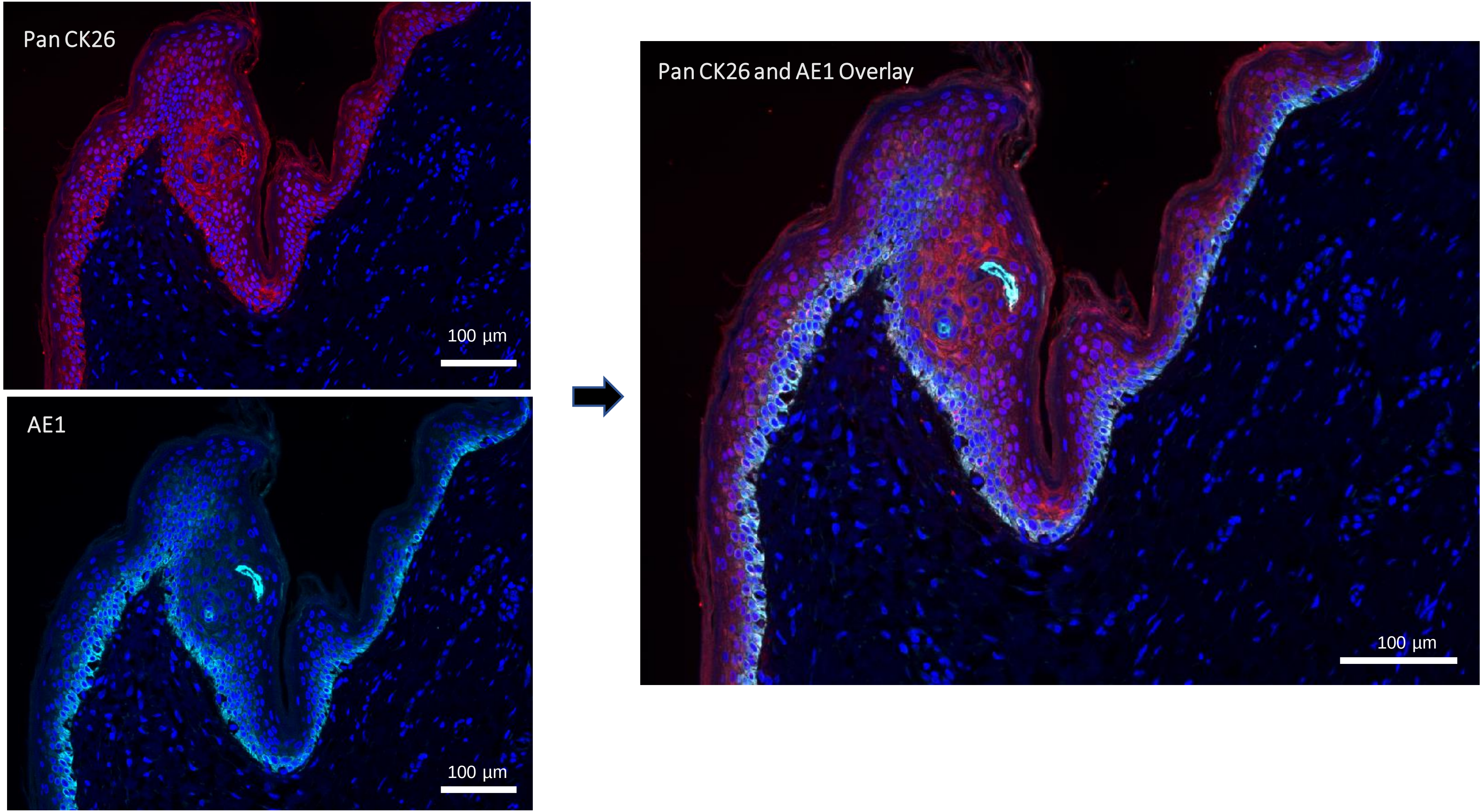

Supplementary Figure 11 - images for KI67 from earlier validation studies and in the multiplexed skin study. As described in methods, a multi-step process was utilized and is available on protocols.io [Cell DIVE™ Platform | Antibody Characterization for Multiplexing (protocols.io)](). A multi-organ slide (Pantomics MTU391) containing tumor and corresponding uninvolved tissues as controls is used for screening antibodies and testing dye conjugates and concentrations. Example images for Ki67 are shown from lymph node and skin.

C

Ki67 (Abcam ab196907, clone EPR3610

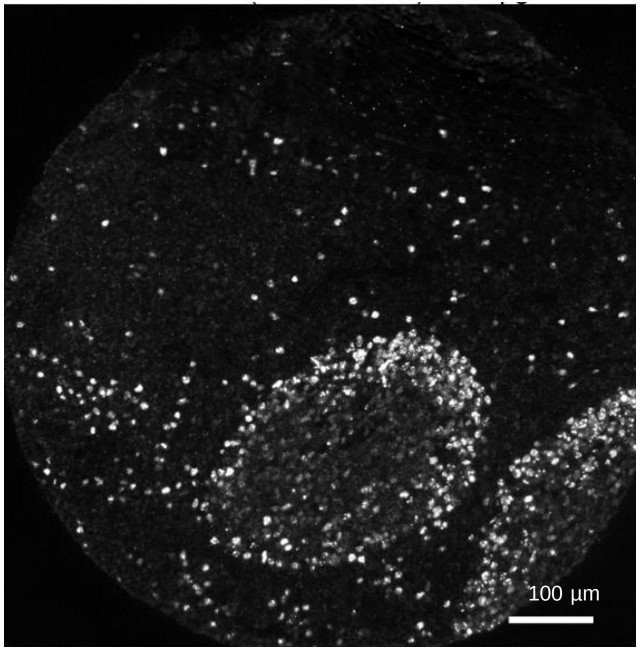

Lymph node – Commercial conjugate Cy5, 10 µg/mL

Region of interest with Ki67 staining in multiplexed skin sample

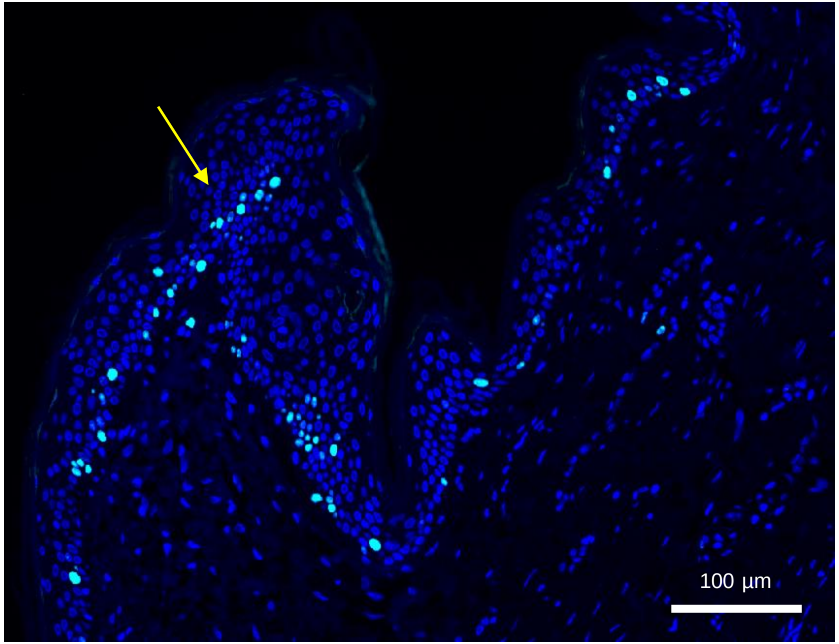

DAPI –Blue, Ki67 - Cyan

Supplementary Figure 11 - images for p53 from earlier validation studies and in the multiplexed skin study. As described in methods, a multi-step process was utilized and is available on protocols.io Cell DIVE™ Platform | Antibody Characterization for Multiplexing (protocols.io). A multi-organ slide (Pantomics MTU391) containing tumor and corresponding uninvolved tissues as controls is used for screening antibodies and testing dye conjugates and concentrations. Example images for p53 are shown from stomach adenocarcinoma and skin.

d

P53 (DAKO M7001, DO-7)

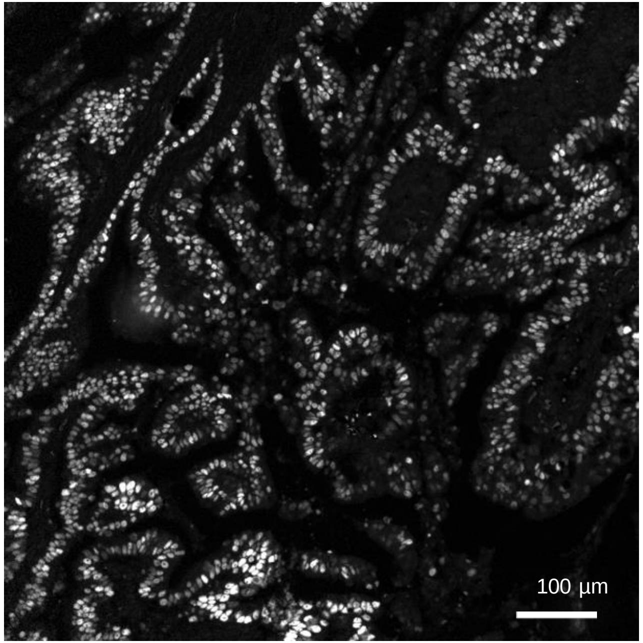

Stomach adenocarcinoma – 1.5 D/P Cy5 at 2 µg/mL

Region of interest with p53 staining in multiplexed skin sample

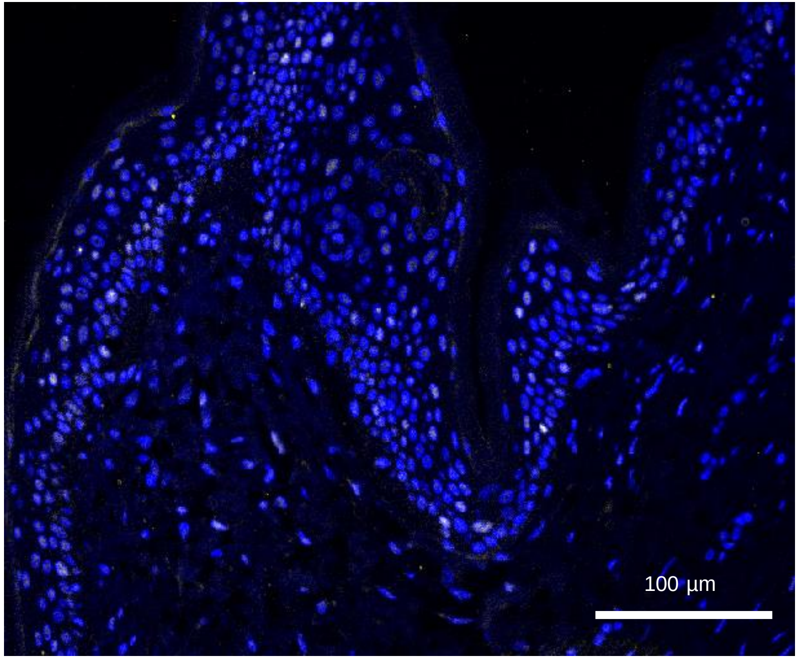

DAPI –Blue, p53 - Yellow

Supplementary Figure 11 - Example images for DDB2 in earlier validation studies and in the multiplexed skin study. As described in methods, a multi-step process was utilized and is available on protocols.io [Cell DIVE™ Platform | Antibody Characterization for Multiplexing (protocols.io)](). A multi-organ slide (Pantomics MTU391) containing tumor and corresponding uninvolved tissues as controls is used for screening antibodies and testing dye conjugates and concentrations. Example images for DDB2 are shown for normal bladder and skin.

e

DDB2 (Abcam ab181136, clone EPR9811)

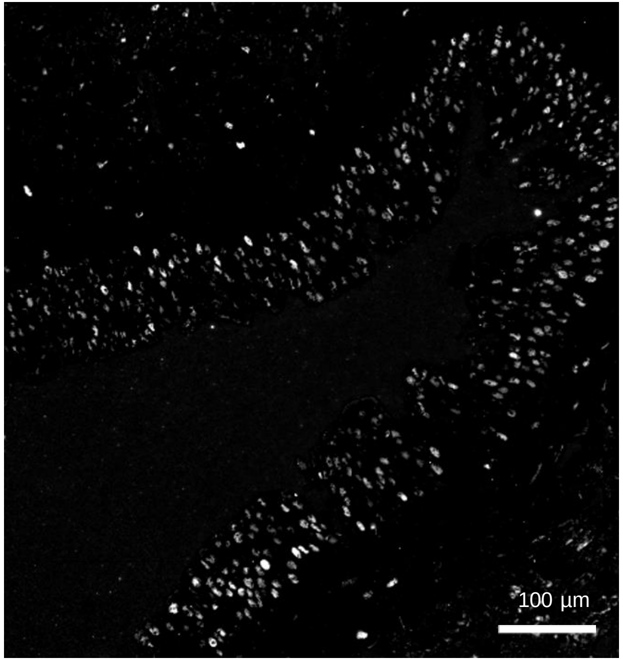

Normal bladder – secondary detection with Cy3 at 6.2 µg/mL

Region of interest with DDB2 staining in multiplexed skin sample

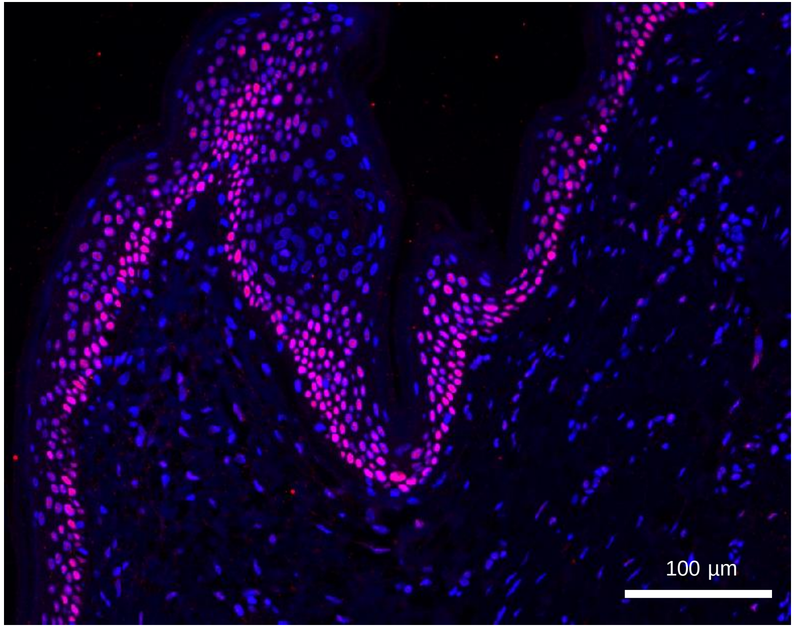

DAPI – Blue, DDB2 - Magenta

Supplementary Figure 11 - Example images for Na+K+ATPase from earlier validation studies and in the multiplexed skin study. As described in methods, a multi-step process was utilized and is available on protocols.io [Cell DIVE™ Platform | Antibody Characterization for Multiplexing (protocols.io)](). A multi-organ slide (Pantomics MTU391) containing tumor and corresponding uninvolved tissues as controls is used for screening antibodies and testing dye conjugates and concentrations. Example images for Na+K+ATPase are shown for breast carcinoma and skin.

f

Na+K+ATPase (Abcam ab167390, clone EP1845Y)

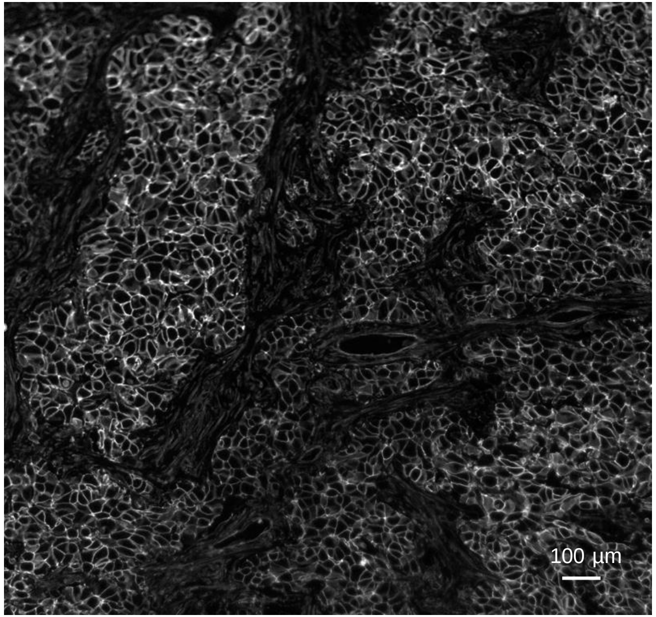

Breast carcinoma –4.2 D/P Cy3 at 5 µg/mL

Region of interest with Na+K+ATPase staining in multiplexed skin sample

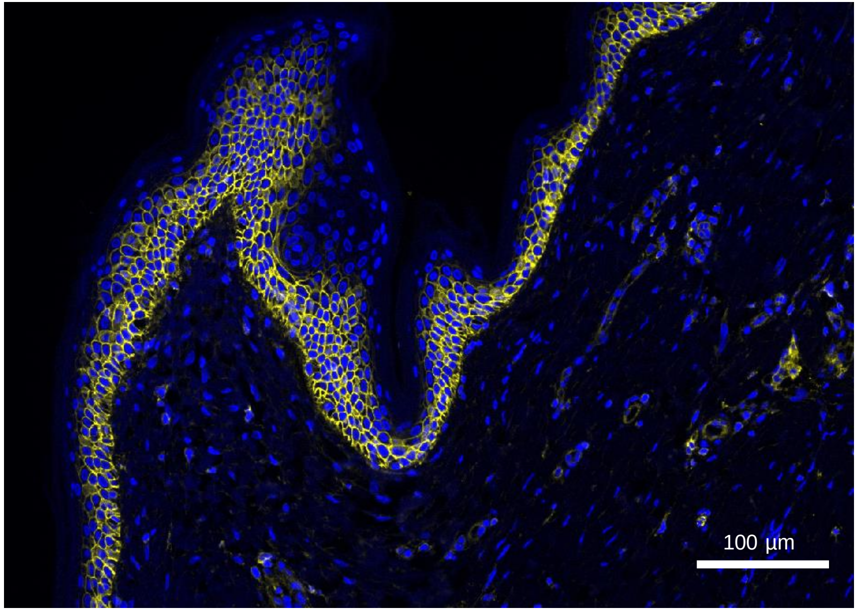

DAPI – Blue, Na+K+ATPase - Yellow

Supplementary Figure 11 - Example images for pan-cadherin from earlier validation studies and in the multiplexed skin study. As described in methods, a multi-step process was utilized and is available on protocols.io Cell DIVE™ Platform | Antibody Characterization for Multiplexing (protocols.io). A multi-organ slide (Pantomics MTU391) containing tumor and corresponding uninvolved tissues as controls is used for screening antibodies and testing dye conjugates and concentrations. Example images for pan-cadherin are shown for normal liver and skin.

g

Pan Cadherin (1-13, 15-20, 22-24) (NeoMarkers, RB-9036, polyclonal antibody)

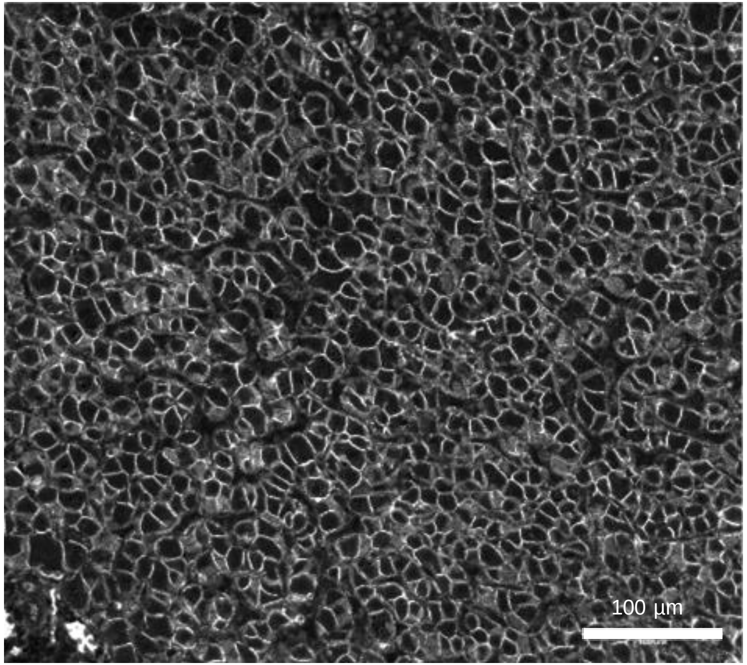

Normal liver – 2.7 D/P Cy5 at 5 µg/mL

Region of interest with pan-cadherin staining in multiplexed skin sample

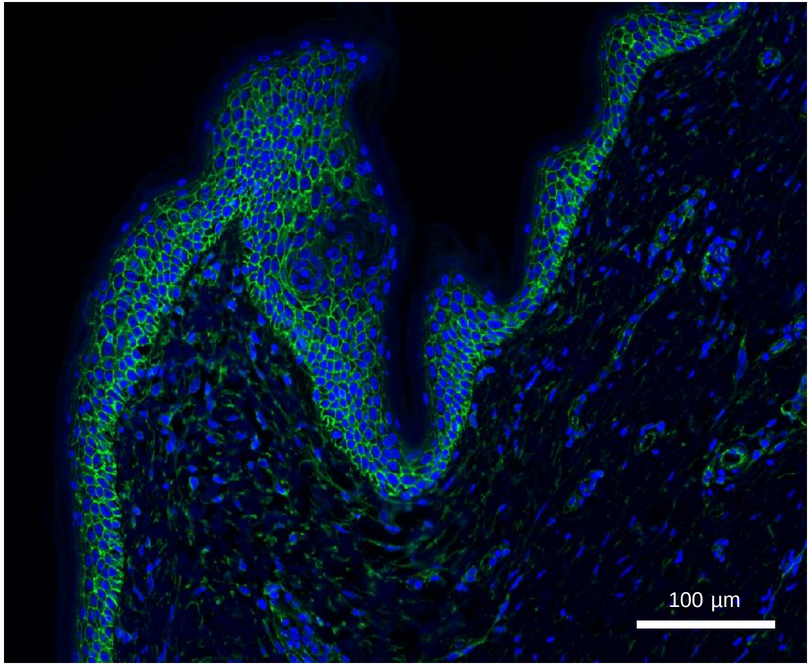

DAPI – Blue, Pan-cadherin - Green

Supplementary Figure 11 - Example images for CD3 from earlier validation studies and in the multiplexed skin study. As described in methods, a multi-step process was utilized and is available on protocols.io [Cell DIVE™ Platform | Antibody Characterization for Multiplexing (protocols.io)](). A multi-organ slide (Pantomics MTU391) containing tumor and corresponding uninvolved tissues as controls is used for screening antibodies and testing dye conjugates and concentrations. Example images for CD3 are shown for breast lymph node and skin.

h

CD3 (DAKO M7254, Clone F7.2.38)

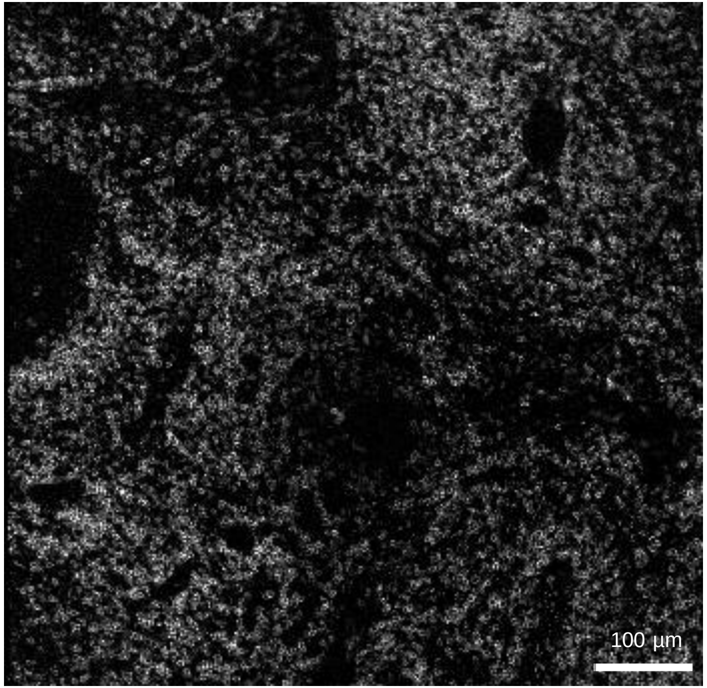

Reactive lymph node 2.5 D/P Cy5 at 5 µg/mL

Region of interest with CD3 staining in multiplexed skin sample

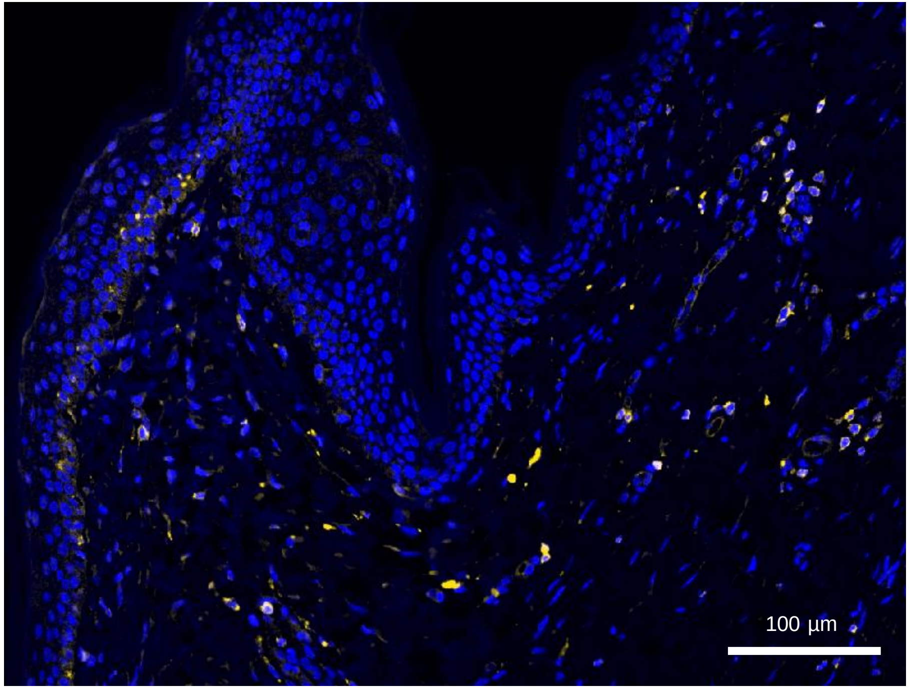

DAPI - Blue, CD3 - Yellow

Supplementary Figure 11 - Example images for CD4 from earlier validation studies and in the multiplexed skin study. As described in methods, a multi-step process was utilized and is available on protocols.io [Cell DIVE™ Platform | Antibody Characterization for Multiplexing (protocols.io)]. A multi-organ slide (Pantomics MTU391) containing tumor and corresponding uninvolved tissues as controls is used for screening antibodies and testing dye conjugates and concentrations. Example images for CD4 are shown for lymph node and skin.

i

CD4 (Abcam ab181724, clone EPR6855)

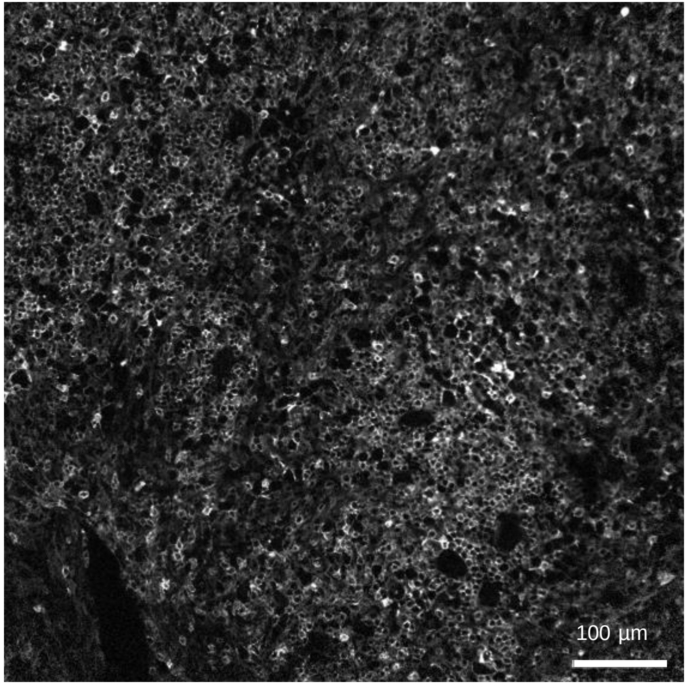

Lymph node, 5.1 D/P Cy3 at 5 µg/mL

Region of interest with CD4 staining in multiplexed skin sample

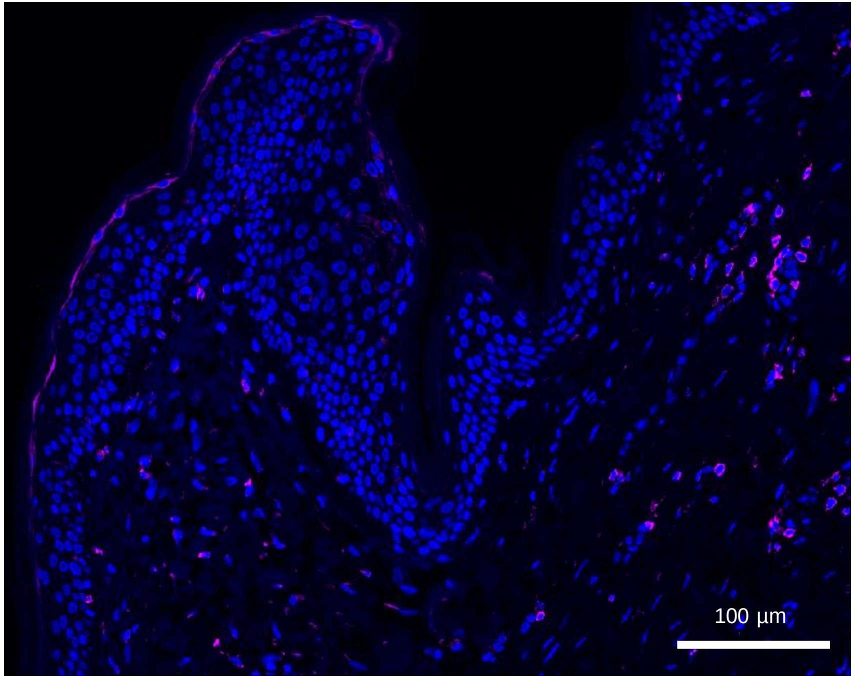

DAPI - Blue, CD4 - Magenta

Supplementary Figure 11 - Example images for FOXP3 from earlier validation studies and in the multiplexed skin study. As described in methods, a multi-step process was utilized and is available on protocols.io Cell DIVE™ Platform | Antibody Characterization for Multiplexing (protocols.io). A multi-organ slide (Pantomics MTU391) containing tumor and corresponding uninvolved tissues as controls is used for screening antibodies and testing dye conjugates and concentrations. Example images for FOXP3 are shown for lymph node and skin.

j

FOXP3 (Biolegend 320114, Clone 206D)

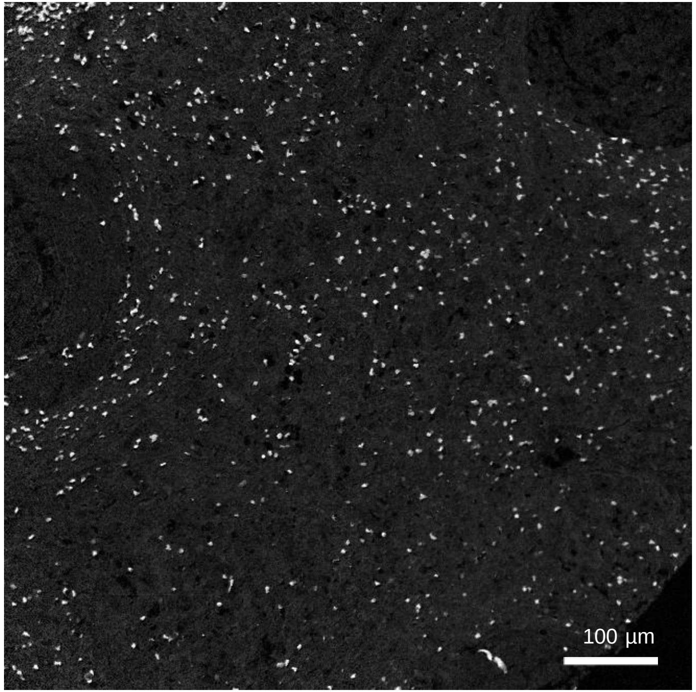

Reactive lymph node, Alexa 647 commercial conjugate, 10 µg/mL

Region of interest with FOXP3 staining in multiplexed skin sample

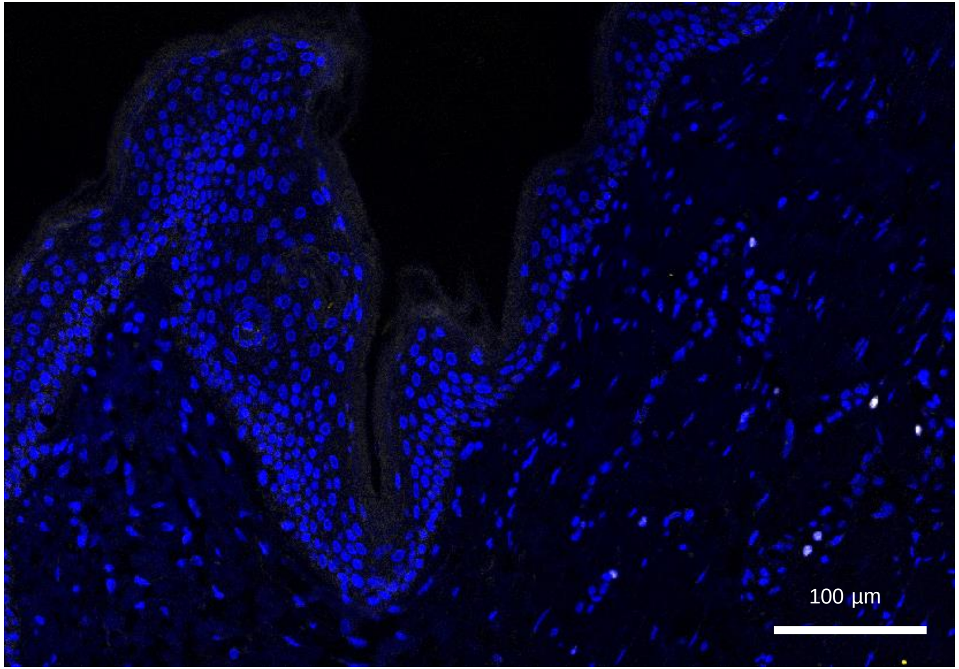

DAPI - Blue, FOXP3 - Yellow

Supplementary Figure 11 - Example images for CD8 from earlier validation studies and in the multiplexed skin study. As described in methods, a multi-step process was utilized and is available on protocols.io [Cell DIVE™ Platform | Antibody Characterization for Multiplexing (protocols.io)](protocols.io). A multi-organ slide (Pantomics MTU391) containing tumor and corresponding uninvolved tissues as controls is used for screening antibodies and testing dye conjugates and concentrations. Example images for CD8 are shown for stomach adenocarcinoma and skin.

k

CD8 (DAKO M7103, Clone C8144B)

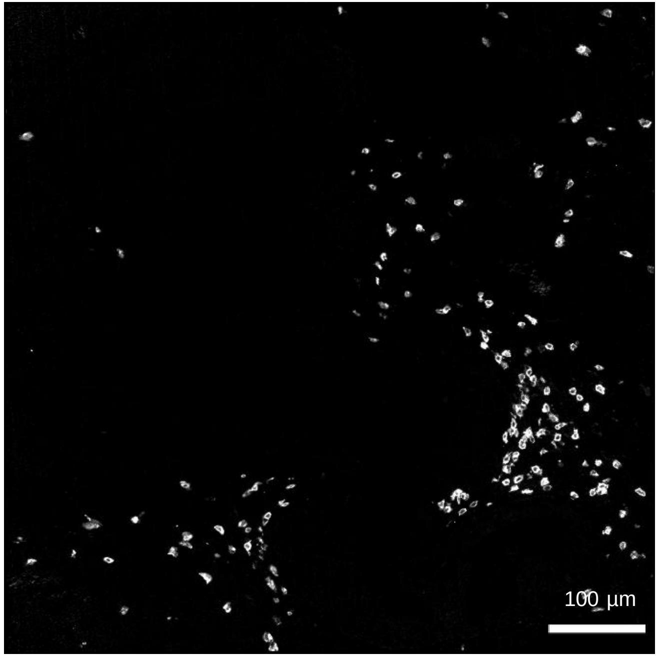

Stomach Adenocarcinoma 2.6 D/P Cy5 at 5 µg/mL

Region of interest with CD8 staining in multiplexed skin sample

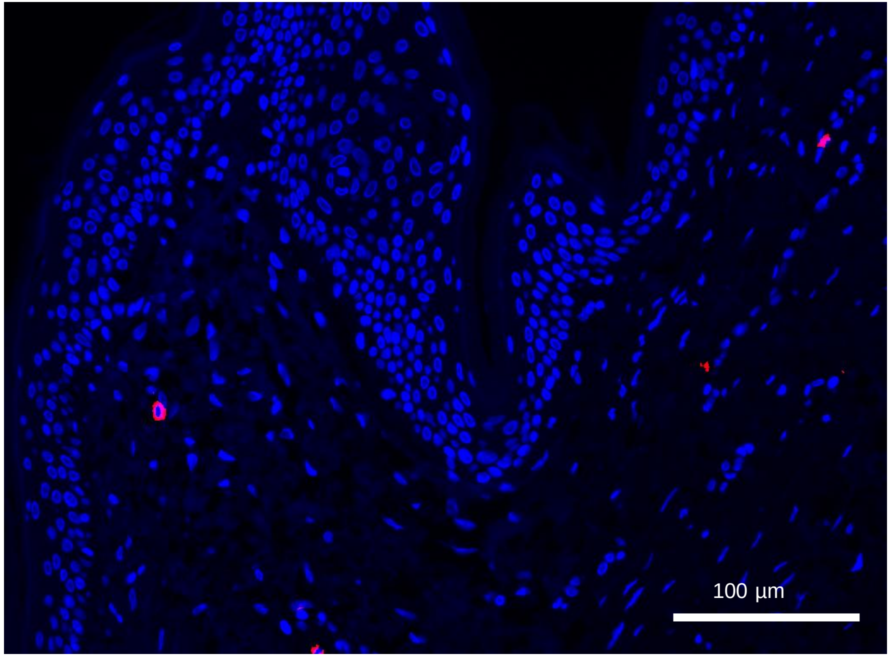

DAPI - Blue, CD8 - Red

Supplementary Figure 11 - Example images for CD68 from earlier validation studies and in the multiplexed skin study. As described in methods, a multi-step process was utilized and is available on protocols.io Cell DIVE™ Platform | Antibody Characterization for Multiplexing (protocols.io). A multi-organ slide (Pantomics MTU391) containing tumor and corresponding uninvolved tissues as controls is used for screening antibodies and testing dye conjugates and concentrations. Example images for CD68 are shown for invasive breast carcinoma and skin.

CD68 (Thermo Fisher MS-397-PABX, clone KP1)

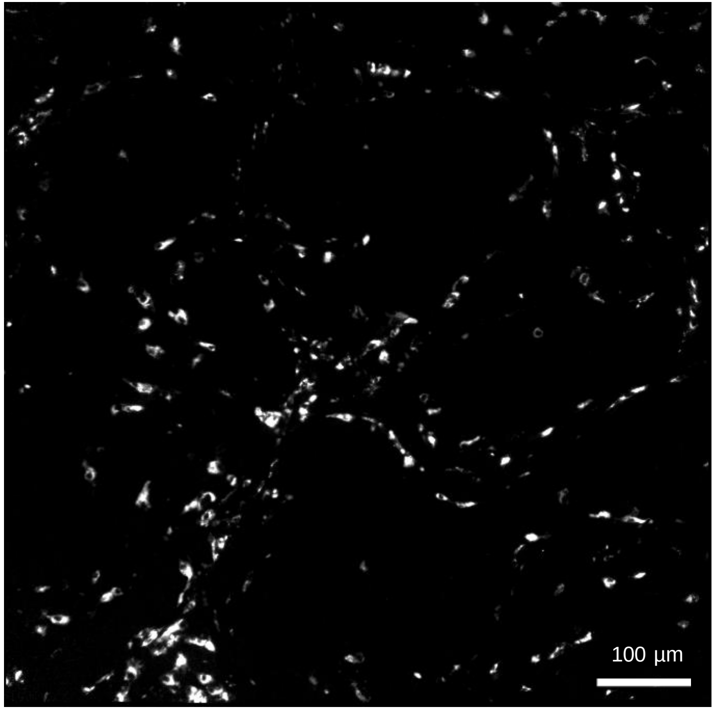

Invasive breast carcinoma 4.0 D/P Cy5 at 3.5 µg/mL

Region of interest with CD68 staining in multiplexed skin sample

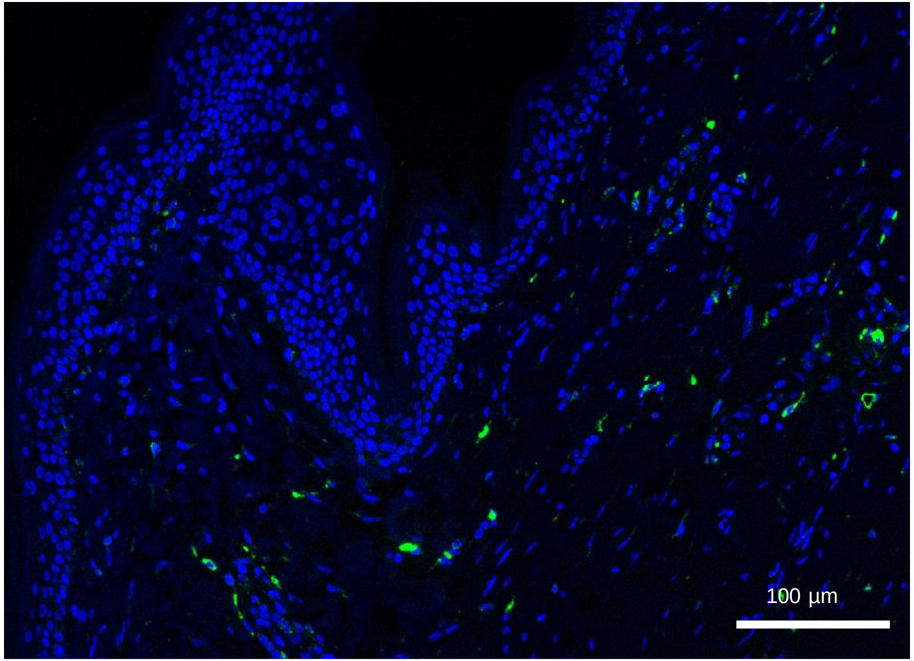

DAPI - Blue, CD68 - Green

Supplementary Figure 11 - Example images for vimentin from earlier validation studies and in the multiplexed skin study. As described in methods, a multi-step process was utilized and is available on protocols.io Cell DIVE™ Platform | Antibody Characterization for Multiplexing (protocols.io). A multi-organ slide (Pantomics MTU391) containing tumor and corresponding uninvolved tissues as controls is used for screening antibodies and testing dye conjugates and concentrations. Example images for vimentin are shown for normal colon and skin.

m **Vimentin (Cell Signaling 9855, clone D21H3)**

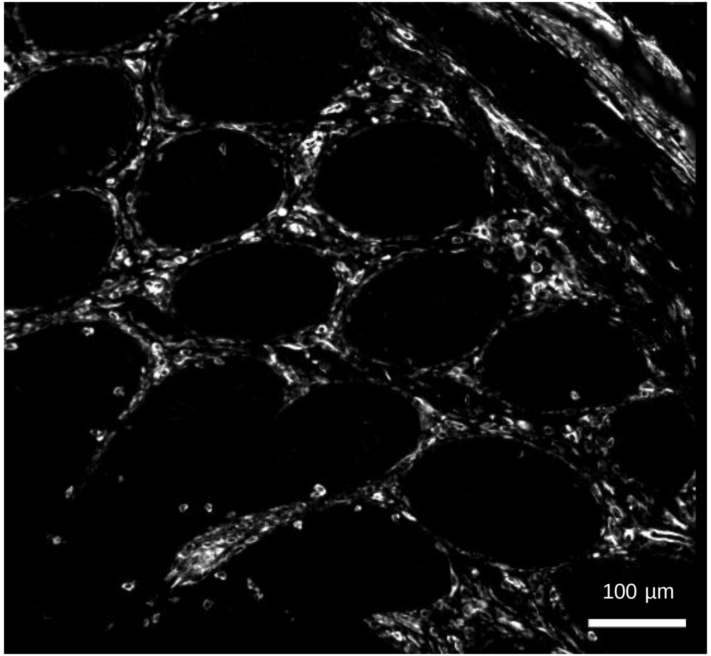

Normal colon – Commercial conjugate Alexa555, at 1.25 µg/mL

Region of interest with Vimentin staining in multiplexed skin sample

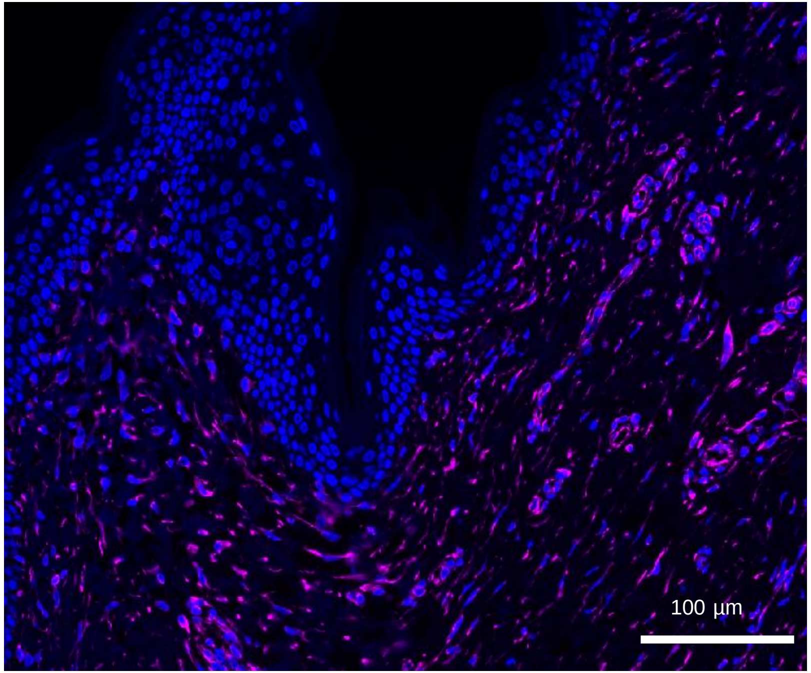

DAPI – Blue, Vimentin - Magenta

Supplementary Figure 11 - Example images for fibronectin from earlier validation studies and in the multiplexed skin study. As described in methods, a multi-step process was utilized and is available on protocols.io Cell DIVE™ Platform | Antibody Characterization for Multiplexing (protocols.io). A multi-organ slide (Pantomics MTU391) containing tumor and corresponding uninvolved tissues as controls is used for screening antibodies and testing dye conjugates and concentrations. Example images for fibronectin are shown for normal pancreas and skin.

n

Fibronectin (Abcam ab206928, Clone F14)

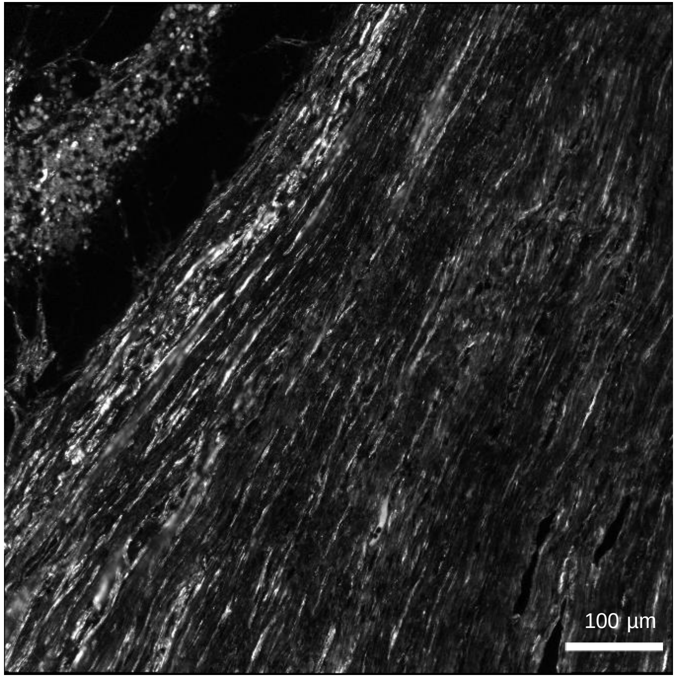

Normal pancreas, 3.4 D/P Cy5 at 5 µg/mL

Region of interest with Fibronectin staining in multiplexed skin sample

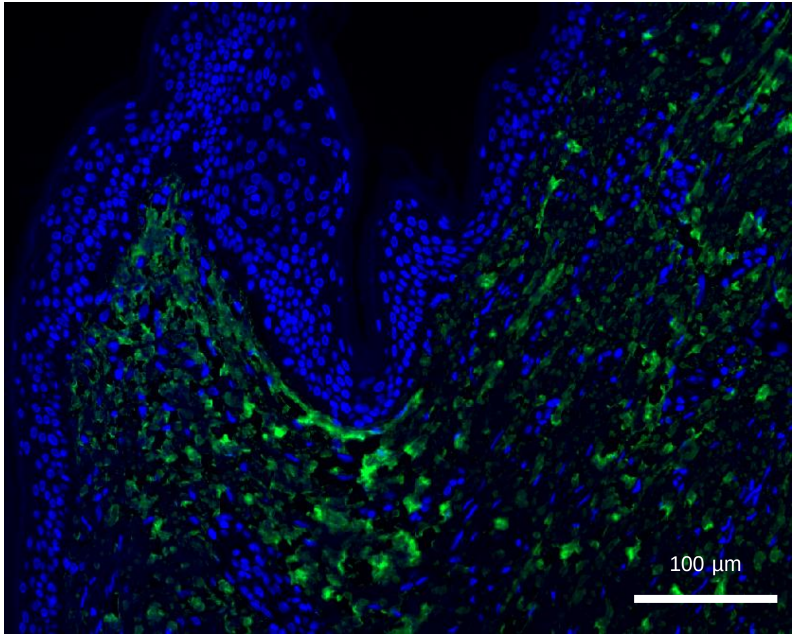

DAPI - Blue, Fibronectin - Green

Supplementary Figure 11 - Example images for CD31 from earlier validation studies and in the multiplexed skin study. As described in methods, a multi-step process was utilized and is available on protocols.io Cell DIVE™ Platform | Antibody Characterization for Multiplexing (protocols.io). A multi-organ slide (Pantomics MTU391) containing tumor and corresponding uninvolved tissues as controls is used for screening antibodies and testing dye conjugates and concentrations. Example images for CD31 are shown for normal colon and skin.

o

CD31 (Cell Signaling 3528BF, Clone 89C2)

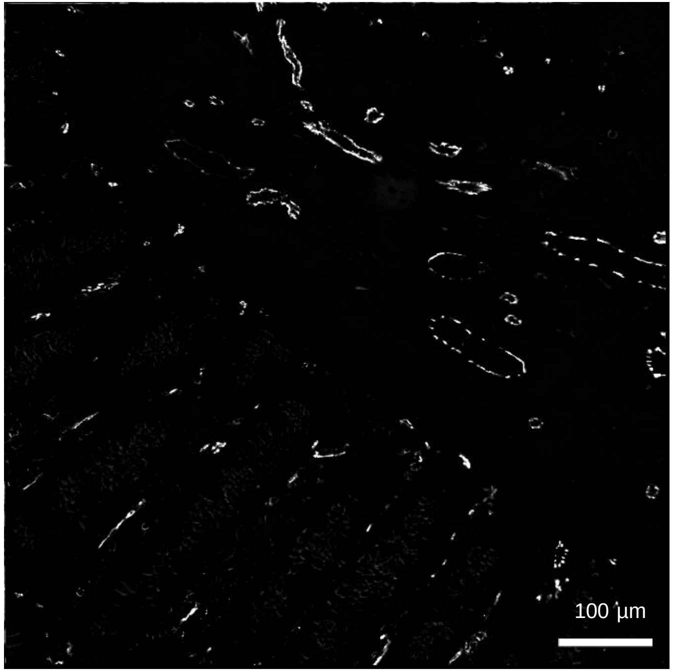

Normal colon, 3.4 D/P Cy3 at 5 µg/mL

Region of interest with CD31 staining in multiplexed skin sample

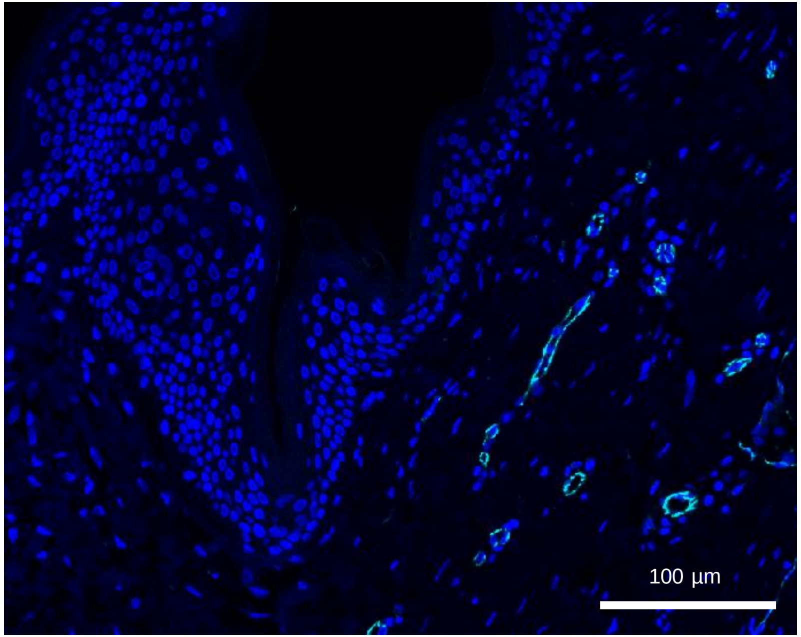

DAPI - Blue, CD31 - Cyan

Supplementary Figure 11 - Example images for collagen IV from earlier validation studies and in the multiplexed skin study. As described in methods, a multi-step process was utilized and is available on protocols.io Cell DIVE™ Platform | Antibody Characterization for Multiplexing (protocols.io). A multi-organ slide (Pantomics MTU391) containing tumor and corresponding uninvolved tissues as controls is used for screening antibodies and testing dye conjugates and concentrations. Example images for collagen IV are shown for normal placenta and skin.

Collagen IV (Millipore MAB3326, clone IV-4H12)

p

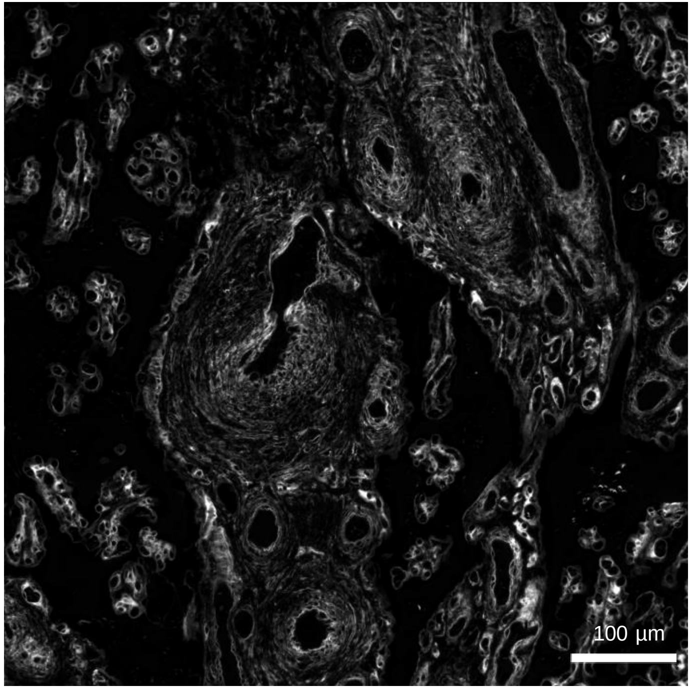

Normal placenta, 3.5 D/P Cy3 at 5 µg/mL

Region of interest with Collagen IV staining in multiplexed skin sample

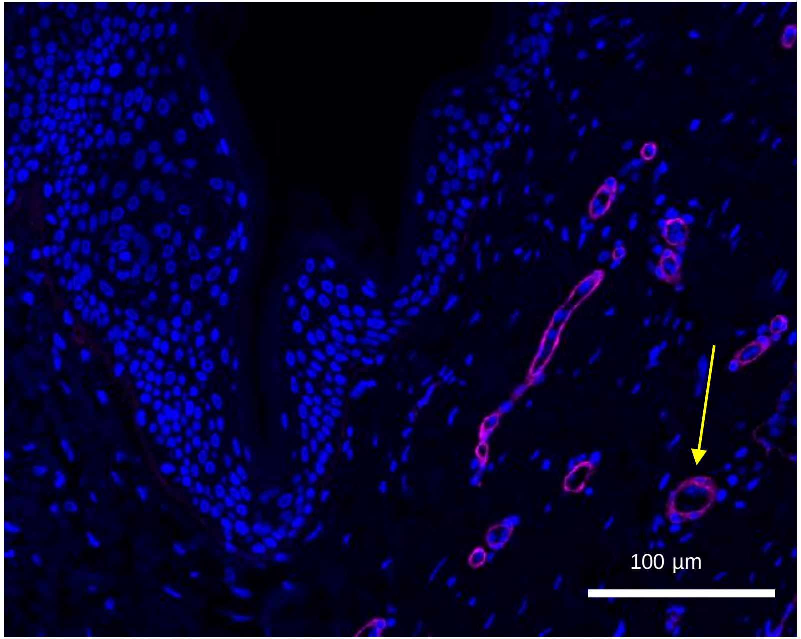

DAPI -Blue, Collagen IV - Magenta

Supplementary Figure 11 - Example images for SMA from earlier validation studies and in the multiplexed skin study. As described in methods, a multi-step process was utilized and is available on protocols.io Cell DIVE™ Platform | Antibody Characterization for Multiplexing (protocols.io). A multi-organ slide (Pantomics MTU391) containing tumor and corresponding uninvolved tissues as controls is used for screening antibodies and testing dye conjugates and concentrations. Example images for SMA are shown for prostate adenocarcinoma and skin.

q

SMA (Sigma C6198, clone 1A4)

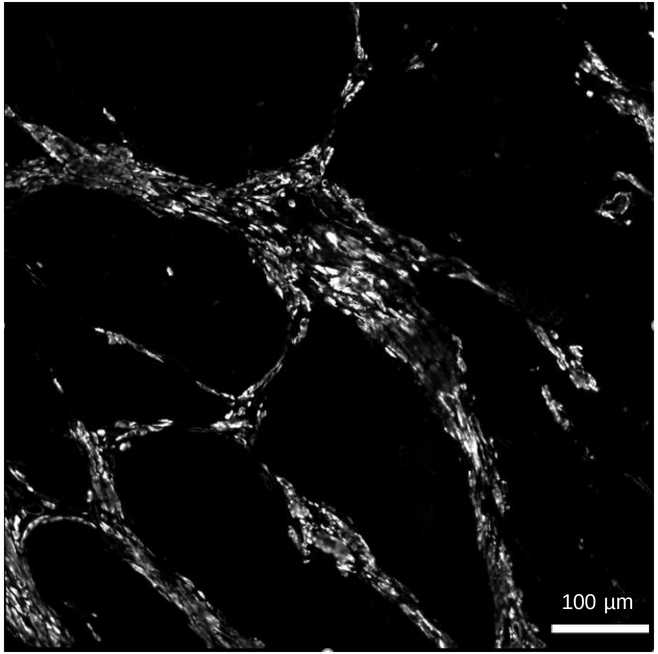

Prostate adenocarcinoma – Commercial conjugate Cy 3, 5 µg/mL

Region of interest with SMA staining in multiplexed skin sample

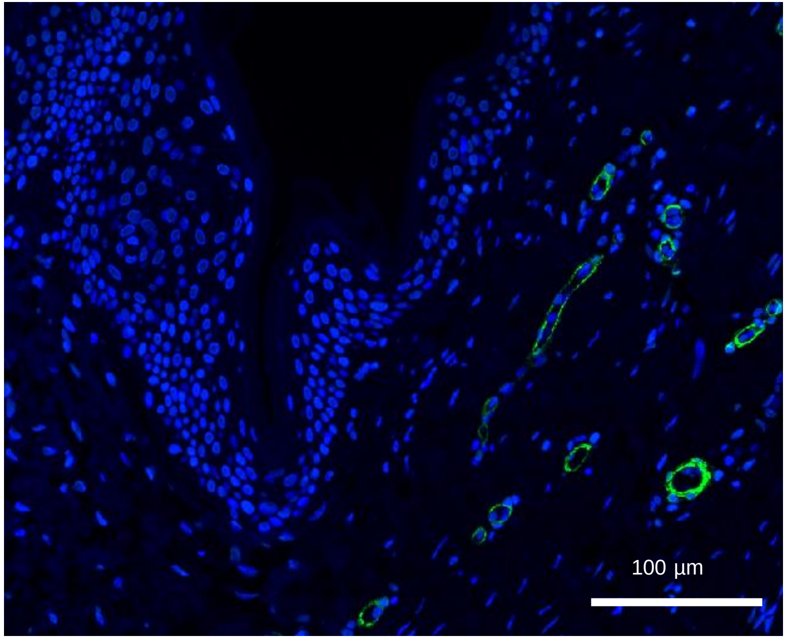

DAPI - Blue, SMA – Green

Supplementary Figure 11 – Shows non-specific staining for UCHL1 antibody. No further analysis was conducted with this data.

r

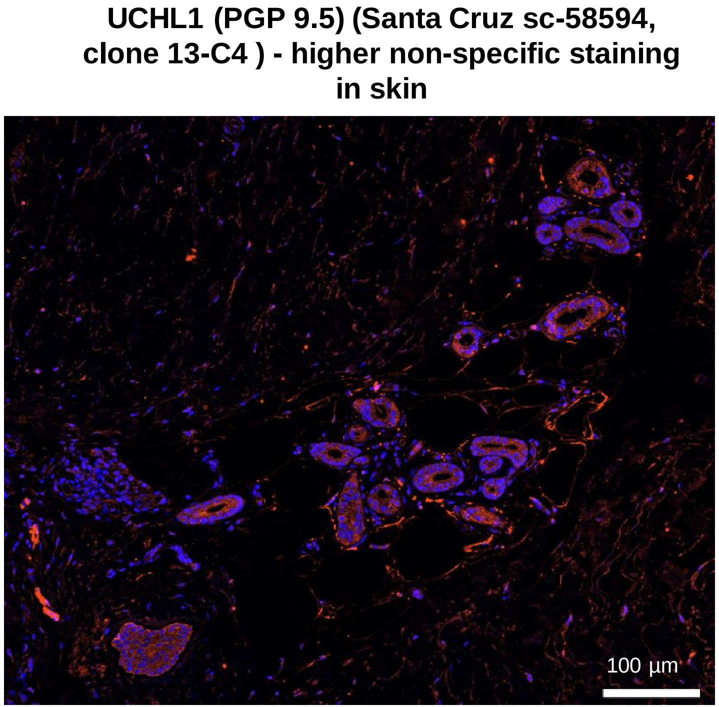

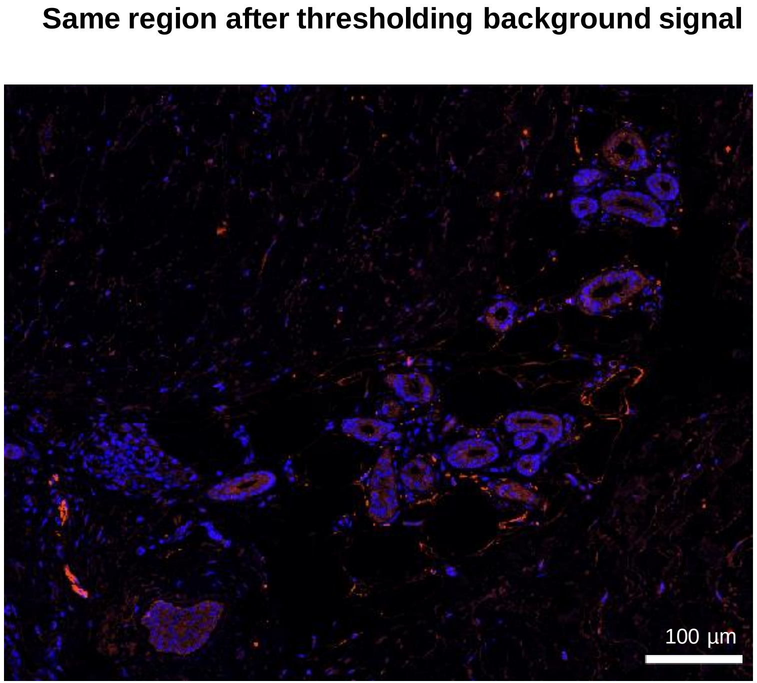

DAPI –Blue, UCHL1 - Red

Supplementary Table 1 Details for donor, sample region, HuBMAP ID (to locate data associated with each sample), anatomical location, sample dimensions (before trimming and embedding), sun exposure effects (based on pathologist review), health status, age, sex, ethnicity of donor.

Sample Region	Donor	HuBMAP ID	Anatomical Location	Dimensions of original sample (cm)	Sun exposure damage in sample by H&E evaluation	Health Status of Donor	Age (years)	Sex	Ethnicity
9	1	HBM372.JZTV.423	L distal upper arm	4.3 x 2.0 x 0.8	Marked	No major illness	60	M	Caucasian
8	2	HBM892.LCXT.493	R arm	3.7 x 2.4 x 0.5	Marked	No major illness; HIV	57	M	Caucasian
1	3	HBM643.WWWB.264	scalp	2.8 x 1.1 x 0.7	Marked	Rheumatoid Arthritis; multiple SCC	72	M	Caucasian
10	4	HBM262.JGDG.897	L distal lower leg	3.0 x 1.3 x 0.7	Moderate	No major illness	53	F	Caucasian
7	5	HBM384.NNQH.676	L lower arm	1.4 x 1.2 x 0.6	Marked	No major illness	69	M	Caucasian
2	6	HBM788.DGPQ.568	L lower mid back	3.6 x 1.7 x 1.3	Mild	No major illness	52	M	Caucasian
11	7	HBM875.KTPB.893	R upper arm	1.7 x 1.2 x 0.9	Mild	No major illness	41	F	Caucasian
6	8	HBM579.CXHK.929	L back	4.2 x 1.5 x 0.8	Excluded	No major illness	35	F	Caucasian
3	9	HBM845.MJNG.266	abdomen	2.8 x 1.2 x 0.7	Mild	No major illness	38	F	Unknown
12	10	HBM636.ZSVF.869	R flank	4.7 x 2.1 x 1.6	Excluded	Systemic lupus erythematosus	32	F	Caucasian
5	11	HBM499.VJVR.265	L abdomen	2.3 x 1.4 x 1.1	Mild	No major illness	33	F	Caucasian
4	12	HBM629.MBTF.679	R superior abdomen	2.1 x 1.1 x 0.6	Mild	No major illness	48	M	Caucasian

Supplementary Table 2 **a-c** Anatomical structure, cell types and biomarkers included in the skin ASCT+B table; **d** biomarkers used in this study. AE1 and CK26 were included as antibody cocktails (keratins are listed in parentheses and specific keratin for a particular cell type is underlined).

Skin Layer	a. Anatomical structure	b. Cell Types	c. Skin biomarkers - version 1.2 Skin ASCT+B Table	d. Biomarkers used in this study
Epidermis	**Stratum corneum**	Keratinocytes (corneocyte)	Involucrin, filaggrin	PCK26 (**KRT1**, KRT5, KRT6, KRT8)
	**Stratum granulosum**	Keratinocytes (Granular)	KRT10	AE1 (**KRT10**, KRT14, KRT15, KRT16 and KRT19), **p53 , MKI67, DDB2**
		Langheran cells	CD1a, CD207, CIITA	-
	**Stratum spinosum**	Prickle cells	KRT1, KRT10	AE1 (**KRT10**, KRT14, KRT15, KRT16 and KRT19), PCK26 (**KRT1**, KRT5, KRT6, KRT8), **p53 , MKI67, DDB2**
		Langheran cells	CD1a, CD207	-
		Merkel cells	KRT20, synaptophysin	-
	**Stratum Basale**	Basal Keratinocytes	KRT5, KRT14	PCK26 (KRT1, **KRT5**, KRT6, KRT8), AE1 (KRT10, **KRT14**, KRT15, KRT16 and KRT19), **p53, MKI67, DDB2**
		Langheran cells	CD1a, CD207	-
		Merkel cells	KRT20, synaptophysin	-
		Melanocytes	SOX10, S100A2, melanA	-
Dermis	**Hair follicle**	Keratinocyte stem cell	KRT15, CD200, CD34	AE1 (KRT10, KRT14, **KRT15**, KRT16 and KRT19)
	**Sebaceous gland**	Sebocyte	MUC1, perilipin 2	-
	**Eccrine gland**	Eccrine cell, myoepithelial cell	KRT19, KRT26	AE1 (KRT10, KRT14, KRT15, KRT16 and **KRT19**)
	**Apocrine glands**	Apocrine cell, myoepithelial cell	GCDFP-15, GATA3	-
		Fibroblasts	Factor XIIIa (dermal dendrocytes with fibroblast cell lineage), vimentin, CD34	**Vimentin**
		Dendritic cells	CD103, CD11b	-
		Macrophages	CD68, CD163	**CD68**
		T helper	CD3D, CD4	**CD3D, CD4**
		T killer	CD3D, CD8	**CD3D, CD8**
		T reg	CD3D, CD4, FOXP3	**CD3D, CD4, FOXP3**
		Natural killer cell	CD3, CD56, CD16	-
		B cells	CD19, CD79	-
		Neutrophils	MPO, p43	-
		Neuron	UCHL1 (PGP 9.5), CRCP	**UCHL1 (PGP 9.5)**
		Endothelial calls	PECAM (CD31), ERG	**PECAM (CD31)**
		Pericyte	SMA, PDGFRB, MCAM	**SMA**
		Lymphatic cells	LYVE1	-

Supplementary Table 3 Antibody, dye and staining information for each marker included in the study

Target Name	Cell type/structure	Clone	Vendor	Catalog Number	Staining concentration (μg/mL)	Conjugate	RRID	Example validation images
**Pan cytokeratin (1, 5, 6A, 6B, 6C, 8)**	Keratinocytes	PCK-26	Sigma	C5992	2.5	Cy3	AB_476824	Supp. Figure 11a
**Pan cytokeratin (10, 14, 15, 16, 19)**	Keratinocytes	AE1	Thermo Fisher	14-9001	2.5	Cy3	AB_1834477	Supp. Figure 11b
**Ki67**	Cell proliferation	SP6	Abcam	ab197547	5	Cy5	AB_60162	Supp. Figure 11c
**p53**	DNA damage	DO-7	Dako	M7001	5	Cy5	AB_2206626	Supp. Figure 11d
**DDB2**	DNA repair	EPR9811	Abcam	ab181136	6.2	Alexa647 secondary	AB_2889873	Supp. Figure 11e
**NaKATPase**	Cell membrane	EP1845Y	Abcam	ab167390	5	Cy3	AB_991679	Supp. Figure 11f
**pan Cadherin (1-13, 15-20, 22-24)**		polyclonal	NeoMarkers	RB-9036	5	Cy5	AB_149846	Supp. Figure 11g
**CD3**	Lymphocytes	F7.2.38	Dako	M7254	5	Cy5	AB_2631163	Supp. Figure 11h
**CD4**	T Helper	EPR6855	Abcam	ab181724	5	Cy3B	AB_2864377	Supp. Figure 11i
**FoxP3**	T Reg	206D	Biolegend	320114	10	Alexa647	AB_439754	Supp. Figure 11j
**CD8**	T killer	C8/144B	Dako	M7103	5	Cy5	AB_2075537	Supp. Figure 11k
**CD68**	Macrophages	KP1	Thermo Fisher	MS-397-PABX	2.5	Cy5	AB_720551	Supp. Figure 11l
**Vimentin**	Fibroblasts	D21H3	Cell Signaling	9855	1.25	Alexa647	AB_10834530	Supp. Figure 11m
**Fibronectin**	Extracellular matrix	F14	Abcam	ab206928	5	Cy5	AB_732380	Supp. Figure 11n
**CD31 (PECAM)**	Endothelial cells	89C2	Cell Signaling	3528BF	5	Cy3	AB_2160882	Supp. Figure 11o
**Collagen IV**	Vessel wall	IV-4H12	Millipore	MAB3326	5	Cy3	AB_2229703	Supp. Figure 11p
**SMA**	Pericytes	1A4	Sigma	C6198	2.5	Cy3	AB_476856	Supp. Figure 11q
**UCHL1 (PGP 9.5)**	Neuron	13C4	Santa cruz	sc-58594	4	Cy3 secondary	AB_793620	Supp. Figure 11r

Supplementary Table 4 T cell counts in 3D reconstructed volumes

Region	Anatomical region	Sun exposure	Voxel count of embedded tissue sample	T cell count in 3D reconstructed volume (24 sections)	volume (um3)	T cell count/um3	Estimated T Cell count/cm3
2	L lower midback	Mild	8111596	1005	40557980	0.000025	24,779,341
3	Abdomen	Mild	9717796	965	48588980	0.000020	19,860,470
4	R superior abdomen	Mild	10559269	2243	52796345	0.000042	42,484,001
5	L abdomen	Mild	8096305	1513	40481525	0.000037	37,375,074
11	R upper arm	Mild	12380681	1168	61903405	0.000019	18,868,106
Mean				***1379***			***28,673,399***
SD				***529***			***10,670,369***
1	Scalp	Marked	5539694	425	27698470	0.000015	15,343,808
7	L lower arm	Marked	5664437	1329	28322185	0.000047	46,924,346
8	R arm	Marked	6966639	1065	34833195	0.000031	30,574,284
9	L distal upper arm	Marked	5454960	1822	27274800	0.000067	66,801,590
10	L distal lower leg	Moderate	5061144	821	25305720	0.000032	32,443,258
Mean				***1092***			***38,417,457***
SD				***526***			***19,414,079***

Supplementary Table 5 DICOM Header for the micro CT images

Tag	Attribute	Value	VR	Length
(0008,0008)	ImageType	[2] ORIGINAL, PRIMARY	CS	16
(0008,0016)	SOPClassUID	1.2.840.10008.5.1.4.1.1.2	UI	26
(0008,0018)	SOPInstanceUID	1.2.840.10008.5.1.4.1.1.2.23942808975273856222255493834451655930	UI	64
(0008,0020)	StudyDate	20200311	DA	8
(0008,0021)	SeriesDate	20200311	DA	8
(0008,0022)	AcquisitionDate	20200311	DA	8
(0008,0030)	StudyTime	132753.000000	TM	14
(0008,0031)	SeriesTime	132753.000000	TM	14
(0008,0032)	AcquisitionTime	132753.000000	TM	14
(0008,0060)	Modality	CT	CS	2
(0008,0070)	Manufacturer	GE	LO	2
(0008,1030)	StudyDescription	IRB-191120023 90kV200uA333ms16um62min	LO	38
(0008,103e)	SeriesDescription	IRB-191120023 90kV200uA333ms16um62min	LO	38
(0008,1070)	OperatorsName	212788497	PN	10
(0010,0020)	PatientID	0	LO	2
(0018,0050)	SliceThickness	0.0160072	DS	10
(0018,0060)	KVP	90	DS	2
(0018,1020)	SoftwareVersions	DICONDE06	LO	10
(0018,1110)	DistanceSourceToDetector	811.425600	DS	10
(0018,1111)	DistanceSourceToPatient	64.943077	DS	10
(0018,1151)	XRayTubeCurrent	200	IS	4
(0018,1160)	FilterType	Unknown	SH	8
(0018,7006)	DetectorDescription	0.016007\0.016007	LT	18
(0018,7020)	DetectorElementPhysicalSize	[2] 0.200000, 0.200000	DS	18
(0020,000d)	StudyInstanceUID	1.2.840.10008.5.1.4.1.1.2.84882527690024139621408019339382376051	UI	64
(0020,000e)	SeriesInstanceUID	1.2.840.10008.5.1.4.1.1.2.22967000708048043635986835612644175816	UI	64
(0020,0010)	StudyID	1	SH	2
(0020,0011)	SeriesNumber	1	IS	2
(0020,0012)	AcquisitionNumber	1	IS	2
(0020,0013)	InstanceNumber	784	IS	4
(0020,0032)	ImagePositionPatient	[3] 0, 0, 12.5496096611023	DS	20
(0020,0037)	ImageOrientationPatient	[6] 1, 0, 0, 0, 1, 0	DS	12
(0028,0002)	SamplesPerPixel	1	US	2
(0028,0004)	PhotometricInterpretation	MONOCHROME2	CS	12
(0028,0010)	Rows	1066	US	2
(0028,0011)	Columns	1999	US	2
(0028,0030)	PixelSpacing	[2] 0.016007, 0.016007	DS	18
(0028,0100)	BitsAllocated	16	US	2
(0028,0101)	BitsStored	16	US	2
(0028,0102)	HighBit	15	US	2
(0028,0103)	PixelRepresentation	1	US	2
(7fe0,0010)	PixelData	2a64	OW	4261868
