# Supplementary material for: 3D reconstruction of skin and spatial mapping of immune cell density, vascular distance and effects of sun exposure and aging
Source: Commun Biol. 2023 Jul 19;6:718. doi: 10.1038/s42003-023-04991-z (PMC10356782; doi:10.1038/s42003-023-04991-z)
Supplement: Supplementary file 3 — Description of Additional Supplementary Files [file 42003_2023_4991_MOESM3_ESM.pdf]

Description of Additional Supplementary Files

File name: Supplementary Data 1
Description: Source data for Figures 4C-E; Figures 5C-D; Figure 6 A-C. Supplementary Figure 8A-C.
