# Supplementary material for: 3D reconstruction of skin and spatial mapping of immune cell density, vascular distance and effects of sun exposure and aging
Source: Commun Biol. 2023 Jul 19;6:718. doi: 10.1038/s42003-023-04991-z (PMC10356782; doi:10.1038/s42003-023-04991-z)
Supplement: Supplementary file 5 — Reporting Summary [file 42003_2023_4991_MOESM5_ESM.pdf]

nature portfolio

Corresponding author(s): DBPR COMMSBIO-22-1401A-Z

Last updated by author(s): 04/12/2023

Reporting Summary

Nature Portfolio wishes to improve the reproducibility of the work that we publish. This form provides structure for consistency and transparency in reporting. For further information on Nature Portfolio policies, see our Editorial Policies and the Editorial Policy Checklist.

Statistics

For all statistical analyses, confirm that the following items are present in the figure legend, table legend, main text, or Methods section.

n/a	Confirmed	
☐	☒	The exact sample size (*n*) for each experimental group/condition, given as a discrete number and unit of measurement
☐	☒	A statement on whether measurements were taken from distinct samples or whether the same sample was measured repeatedly
☐	☒	The statistical test(s) used AND whether they are one- or two-sided<br>*Only common tests should be described solely by name; describe more complex techniques in the Methods section.*
☐	☒	A description of all covariates tested
☐	☒	A description of any assumptions or corrections, such as tests of normality and adjustment for multiple comparisons
☐	☒	A full description of the statistical parameters including central tendency (e.g. means) or other basic estimates (e.g. regression coefficient) AND variation (e.g. standard deviation) or associated estimates of uncertainty (e.g. confidence intervals)
☐	☒	For null hypothesis testing, the test statistic (e.g. *F*, *t*, *r*) with confidence intervals, effect sizes, degrees of freedom and *P* value noted<br>*Give P values as exact values whenever suitable.*
☒	☐	For Bayesian analysis, information on the choice of priors and Markov chain Monte Carlo settings
☒	☐	For hierarchical and complex designs, identification of the appropriate level for tests and full reporting of outcomes
☐	☒	Estimates of effect sizes (e.g. Cohen's *d*, Pearson's *r*), indicating how they were calculated

Our web collection on statistics for biologists contains articles on many of the points above.

Software and code

Policy information about availability of computer code

Data collection	Samples were imaged and processed using Cell DIVE software (Leica Microsystems); images were processed using cell segmentation and 3D reconstruction software developed specifically for this study. All code can be found at GitHub - hubmapconsortium/MATRICS-A: Multiplexed Image Three-D Reconstruction and Integrated Cell Spatial -Analysis (https://github.com/hubmapconsortium/MATRICS-A). There is a corresponding ReadMe file that provides context and instructions for the repository’s contents and could be found here MATRICS-A/ README.md at main · hubmapconsortium/MATRICS-A · GitHub. The docker container/environment to run the code can obtained by using docker pull hubmap/gehc:skin. and test data for skin region 7 can be found at Human Digital Twin: Automated Cell Type Distance Computation and 3D Atlas Construction in Multiplexed Skin Biopsies | Zenodo. Multiple opensource software were used to cerate the docker container environment and build the code including ITK (ver 5.1), Tensorflow (ver 1.15), Keras (ver 2.2.4), opencv-python (ver 3.4), tifffile (ver 2020.9), Nifty-Reg (ver 1.3), Python (ver 3.6), CMake (ver 3.17). We highly recommend using the docker container to run the code.
Data analysis	Statistical analysis was conducted using R version 4.1.2, and additional packages reshape (0.8.9), ggplot2 (3.3.6), and plyr (1.8.7) were used for data processing and visualization.

For manuscripts utilizing custom algorithms or software that are central to the research but not yet described in published literature, software must be made available to editors and reviewers. We strongly encourage code deposition in a community repository (e.g. GitHub). See the Nature Portfolio guidelines for submitting code & software for further information.

Data

Policy information about availability of data

All manuscripts must include a data availability statement. This statement should provide the following information, where applicable:

- Accession codes, unique identifiers, or web links for publicly available datasets
- A description of any restrictions on data availability
- For clinical datasets or third party data, please ensure that the statement adheres to our policy

The anonymized data that support the findings of this study are included in Zenodo https://zenodo.org/record/7565670#.ZDbF_ObMIuV (Supplementary Data 1 - source data for Figures 4C-E; Figures 5C-D; Figure 6 A-C. Supplementary Figure 8A-C) or available as a downloadable file from this paper - Supplementary Data 2 - source data for Figure 5A; Supplementary Figures 6, 7 and 9. All original images for each donor/region/sequential section are available via publicly accessible HuBMAP Globus sites for each donor/region https://hubmapconsortium.github.io/vccf-visualization-2022/.

Human research participants

Policy information about studies involving human research participants and Sex and Gender in Research.

Reporting on sex and gender	This study included 6 males and 6 females, data from 2 females was excluded due to skin pathologies. The final data set was 6 males and 4 females. We did not conduct statistical analysis based on sex due to small sample size.
Population characteristics	Skin biopsies were collected from 12 donors ranging from 32-72 years with a mix of typically UV-exposed and non-exposed anatomical regions. Of the 12 samples, 10 were down selected for further statistical analysis. The two excluded samples included a donor with a benign cyst, but with extensive inflammation and immune cell infiltration compared to other samples (region 6). The second sample had a scar which also altered the normal organization of the epidermis and dermis layers (region 12). All donors were in good health and cancer free at the time of sample collection, with two donors having chronic diseases, which is noted in the patient summary table
Recruitment	Samples were retrospective archived samples and selected for inclusion in the study based on donor age and UV exposure.
Ethics oversight	The study protocol was approved by University of Pittsburgh IRB (STUDY19120023).

Note that full information on the approval of the study protocol must also be provided in the manuscript.

Field-specific reporting

Please select the one below that is the best fit for your research. If you are not sure, read the appropriate sections before making your selection.

☒ Life sciences ☐ Behavioural & social sciences ☐ Ecological, evolutionary & environmental sciences

For a reference copy of the document with all sections, see nature.com/documents/nr-reporting-summary-flat.pdf

Life sciences study design

All studies must disclose on these points even when the disclosure is negative.

Sample size	As this was an exploratory study for demonstrating 3D reconstruction, no formal sample size was conducted. We selected the 12 samples based on the maximum number of samples that would fit within a single tissue block.
Data exclusions	Of the 12 samples, 10 were down selected for further statistical analysis. The two excluded samples included a donor with a benign cyst, but with extensive inflammation and immune cell infiltration compared to other samples (region 6). The second sample had a scar which also altered the normal organization of the epidermis and dermis layers (region 12).
Replication	The block containing the 12 samples was first sectioned into 100 sections, and 26 of the highest quality sections from each patient were used for multiplexed imaging.
Randomization	Since all samples were embedded into a single tissue block, and were sectioned onto the same slide, and underwent the same staining/ imaging workflow, no additional randomization was conducted.
Blinding	Researchers were not blinded to group (analysis was conducted by age and UV exposure) as the analysis was exploratory and required discussion with the pathologist to interpret findings during data analysis.

Reporting for specific materials, systems and methods

We require information from authors about some types of materials, experimental systems and methods used in many studies. Here, indicate whether each material, system or method listed is relevant to your study. If you are not sure if a list item applies to your research, read the appropriate section before selecting a response.

Materials & experimental systems

n/a	Involved in the study
☐	☒ Antibodies
☒	☐ Eukaryotic cell lines
☒	☐ Palaeontology and archaeology
☒	☐ Animals and other organisms
☒	☐ Clinical data
☒	☐ Dual use research of concern

Methods

n/a	Involved in the study
☒	☐ ChIP-seq
☒	☐ Flow cytometry
☒	☐ MRI-based neuroimaging

Antibodies

Antibodies used	All details for antibodies including supplier name, catalogue number, lot number, clone number are described in supplementary Table 3.
Validation	All antibodies used in this study were subjected to a standardized characterization process and the full protocol is provided on https://www.protocols.io/view/cell-dive-platform-antibody-characterization-for-m-4r3l247rqg1y/v1. We typically start characterization using a reference multiorgan TMA (MTU391, Pantomics) which contains 15 major types of cancer (surgically resected) and corresponding uninvolved tissues as controls. Initial characterization and down-selection includes (1) screening multiple clones/target that are compatible with immunohistochemical detection in FFPE tissue (using published literature and Human Protein Atlas); (2) evaluating performance specificity using the MTU391 array and isotype control using a labeled secondary antibody; 3) to confirm epitope stability to the multiplexed cycling process, unstained MTU391 slides are processed through multiple rounds of signal inactivation and then stained to evaluate whether target intensity had decreased. In this study, none of the epitopes showed sensitivity to the signal inactivation protocol (i.e., staining intensity did not decrease following inactivation); 4) the best performing antibodies were conjugated to a fluorescent dye at multiple dye:protein ratios and titrated on sequential MTU391 TMA sections to compare sensitivity and specificity to the unmodified primary antibody. Primary secondary detection be also used in the first round of staining (assuming different species are available), which provides flexibility for any antibody that cannot be successfully conjugated. For the current study, all antibodies were tested in MTU391 arrays as described and then re-tested in a pilot study using 10 skin samples provided by U. Pitt Dermatopathology department.
